# Monitoring Advanced Hydration Station (AHS) Water Quality in Flint, MI, School Buildings: Field Grab Sampling and Filter-Based Lead Assessment

**DOI:** 10.1021/acs.est.6c09190

**Published:** 2026-10-23

**Authors:** Zehua Wang, Laura Sullivan, Michelle Ammerman, Jean M. Brownell, Anushka Mishrra, Conor Gowan, Shawn P. McElmurry, Daniel E. Giammar, Nancy G. Love

**Affiliations:** † Department of Energy, Environmental and Chemical Engineering, 7548 Washington University in St. Louis, St. Louis, Missouri 63150, United States; ‡ Department of Mechanical Engineering, 3364 Kettering University, Flint, Michigan 48504, United States; § Department of Civil and Environmental Engineering, 1259 University of Michigan, Ann Arbor, Michigan 48109, United States; ∥ Department of Civil and Environmental Engineering, Wayne State University, Detroit, Michigan 48202, United States

**Keywords:** lead exposure, school filters, bacteria, acid-extracted, volume-averaged concentration, filter-based monitoring

## Abstract

School drinking water lead exposure remains a public health concern. In addition, time-variant lead concentrations pose challenges to monitoring practices. This study evaluates the lead (and, for some experiments, bacterial) removal performance of advanced hydration stations (AHS) with membrane filters, activated carbon block filters, and LED UV disinfection in Flint, Michigan. Laboratory challenge testing and more than 400 grab samples were collected from influent and effluent water from over 70 field-deployed AHS units during three sampling campaigns in 2022 and 2023. Our study confirmed that AHS units effectively removed lead. The median influent lead concentrations ranged from 6 to 14 μg/L, with 53% of samples exceeding 10 μg/L. Lead levels in effluent water were almost all below the detection limit (98% of samples). Effluent bacteria concentrations monitored during the first field sampling campaign in 2022 were all below detection, and removal during challenge testing were >2.6 log reduction. A subset of filters (n = 21) from those harvested in July 2022 were acid-extracted to assess the total accumulation of lead. This information, along with the cumulative volume of water processed, was used to calculate a volume-averaged lead concentration that represents avoided lead exposure and identifies potential hot spots of lead release to prioritize remediation activities. The outcomes of this monitoring campaign demonstrate the effectiveness of the AHS units at removing lead and bacteria, and the importance of using filter-based monitoring to inform AHS maintenance and identify upstream sources of lead.

## Graphical abstract

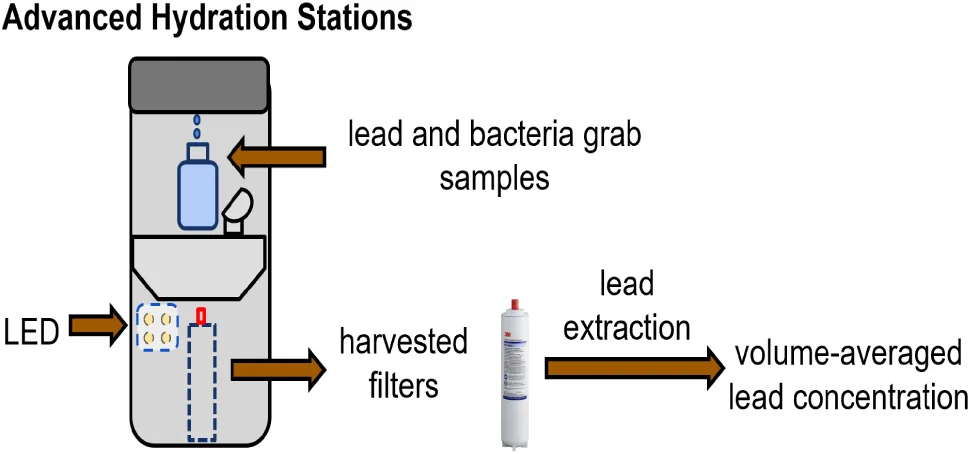

## 1 Introduction

School building infrastructure and its impact on the learning environment directly influence not only children’s health but also their educational outcomes and success.^1–3^ It is for this reason that removal of lead, a potent neurotoxin,^4^ from school building environments is a high priority. The Safe Drinking Water Act Lead Ban of 1986 required the use of “lead-free” materials (8% for pipes and 0.2% for solders and fluxes) for new or reconstructed public buildings.^5^ Over a decade later in 1998, the U.S. Congress required the U.S. Environmental Protection Agency (EPA) to develop protocols and rules around lead abatement in buildings frequented by children, including schools.^6^ According to the Safe Drinking Water Act (2014), “lead-free” pipes, fittings and fixtures are subjected to a more stringent regulation on weighted average from 8% to 0.25% lead.^7^ Despite the intent of these earlier efforts, lead contributions through drinking water have not been removed from all schools. Complicating this is the fact that many plumbing products on the market still contain some lead, especially older products.^8–11^

Lead testing in schools is challenging due to the potential presence of legacy plumbing materials, the spatiotemporal variation of lead and limited federal regulation. Plumbing age in school buildings and associated lead leaching potential are concerning. In 2014, the average age of school buildings in the U.S. was 44 years old, and those in the Northeast and Central U.S. are generally older.^12^ In Flint, Michigan’s public schools, where the work presented here is centered, Flint Community School (FCS) building age averaged 70 years as of 2021.^13^ These old plumbing materials represent a significant source of potential lead contamination. Lead can be present in drinking water in dissolved or particulate forms that respond to changes in water chemistry and hydraulics, including long stagnation periods that frequently occur in schools, given their varied use patterns.^14–18^ Collectively, this can result in episodic lead release events that are difficult to predict.^11,19–21^ Our team’s lens into the problem of school environments that have the potential to negatively impact student learning and livelihoods was brought into focus by the conditions experienced with sequential crises; namely, the Flint Water Crisis (beginning in 2014), followed by school shutdowns due to the COVID-19 pandemic (beginning in 2020). Unfortunately, federal regulations had not required water quality monitoring in schools until the recently published Lead and Copper Rule Improvements (LCRI).^4^ Consequently, the persistence of lead in aging school buildings is not well documented and represents a gap in the literature. While some school districts have voluntarily monitored lead levels in school building drinking water,^22–25^ monitoring was not required and results were not required to be shared, leading to the issue of lead in school drinking water being insufficiently understood.^26^

Some U.S. states have adopted a “test and fix” approach to lead in schools, which requires lead testing followed by mitigation actions if the lead level exceeds regulated thresholds. Lead monitoring practices primarily include taking a grab sample, which uses a fixed-volume water sample taken at a single point in time. However, this reactive approach is deemed risky for two main reasons. First, grab sampling does not provide protection. Users will have already been exposed to lead before grab samples indicate a problem. Second, grab samples are often used to identify which faucets or pipes need remediation, but they should not be used to infer lead exposure. It has been shown that grab samples cannot reliably indicate overall lead exposure, given the spatiotemporal variability of lead levels in drinking water.^27,28^ Many years after its lead crisis, Washington, D C., was the first U.S. jurisdiction to recognize the failings of “test and fix”; instead, the District now requires point-of-use (POU) hydration station (HyS) units (i.e., drinking water bubblers that include bottle fillers) combined with NSF 53-certified lead-removing filters in all schools.^29^ In 2023, Michigan became the first state in the U.S. to pass comprehensive “Filter First” legislation for all schools and childcare centers.^30,31^ The effort by these U.S. jurisdictions to implement large-scale filtration programs in school buildings will likely motivate other states to follow in the future.

The most commonly deployed “lead filters” use activated carbon-block cartridges, which are highly reliable at removing both dissolved and particulate lead under a range of water quality conditions.^32–36^ However, few published studies have been conducted in schools and verified lead removal performance under public use conditions. In prior work, we found that filters effectively removed lead and that some lead was released into stagnant water following overnight stagnation in schools served by groundwater in Illinois.^33^ Despite the reliability of activated carbon block filters for lead removal, the use of filters in schools is not without consequences. More recent studies^37,38^ have corroborated historical studies,^39–41^ affirming that bacterial growth occurs in activated carbon block filters and that bacterial cell counts increase in filtered water. Evidence that non-enteric pathogen growth can occur has been provided through laboratory studies.^37,38^ Bacterial levels can be minimized in filtered water by (1) implementing routine maintenance practices that include flushing^42^ and (2) coupling filtration with disinfection.^43^ Some manufacturers have incorporated silver particles into activated carbon block filters to inhibit bacterial growth; however, silver leaching is a common challenge and the disinfectant capacity of these filters tends to be short-lived.^44,45^ Beyond bacterial growth on the filters, drawbacks of using filtration in schools include the cost of filters and HyS maintenance, particularly for under-resourced school districts. Under Michigan’s “Filter First” legislation, filter replacement frequency is not mandated, but schools must create a Drinking Water Management Plan (DWMP) that includes a replacement-frequency plan for each school building. At this time, the only basis for replacement is the volume of water filtered, as indicated by lights on the front of the units. This indicator has not been shown to correlate with lead removal performance in the field, which can vary widely based on drinking water chemistry and field conditions.^46,47^ Therefore, better indicators are needed to address this gap.

In addition to removing lead, activated carbon block filters used in HyS units can serve as volume-integrated monitoring tools that quantify the total lead entering a filter over its service life. Many hydration stations indicate the cumulative volume of water processed by filters (e.g., number of 0.59-L bottles of water saved), but do not provide water quality information. Volume-averaged lead concentrations can be determined from the volume of water processed by filters and the total mass of lead removed by the filter, as measured by effectively extracting lead from the filter. Previous work has demonstrated that using 1 M nitric acid in a flow-through manner can extract almost all accumulated lead, both in dissolved and particulate forms, from faucet-mounted POU filters,^48–50^ which have similar physical structure and chemical composition to those used in school HyS units.^51,52^ However, this approach had not previously been validated for filters installed in HyS units in real school settings. Employing this procedure with FCS-deployed filters provides an opportunity to collect field-relevant data that starts to fill this gap.

This paper couples the results of an outreach partnership that supported FCS as it initiated use of 75 advanced HyS (AHS) units in 2022 and resulted in the generation of an unprecedented AHS challenge test and field performance data set, along with a detailed comparison of grab sample versus volume-averaged lead concentrations in field-deployed AHS units inside FCS’s aged school buildings. It is the only FCS-approved study of the AHS units installed in the schools in the aftermath of the Flint Water Crisis. The AHS units employed in FCS incorporated two treatment steps for lead removal (membrane and activated carbon block filtration) and disinfection via LED UV light, rendering them more advanced than the average HyS used in public settings. They were acquired prior to the COVID-19 pandemic and underwent laboratory lead and bacterial challenge testing during the pandemic that resulted in design modifications. The installed units were upgraded to incorporate these modifications, and their ability to remove lead and bacteria was verified in the field before they were made available for use in February 2022. Field monitoring continued through October 2023, which focused solely on lead removal performance. This paper presents a unique data set that demonstrates the importance of using high-quality components in AHS units, how those units performed in aged school buildings, and the utility of combining grab and volume-averaged lead measurements to prioritize lead mitigation decisions that can guide schools implementing Filter First programs.

## 2 Materials and Methods

### 2.1 Hydration Station Design

The FCS AHS units originally arrived from the manufacturer with an ultramembrane filter and a separate activated carbon block filter, neither of which was certified to National Sanitation Foundation (NSF) standards. After poor performance during initial laboratory challenge testing, we replaced the two manufacturer filters with a single 3 M FM1500-B filter (5,678 L capacity) that is certified to NSF Standards 42 and 53 for the removal of lead.^51^ This integrated filter contains a concentrically outer 0.2-μm microfilter membrane and a concentrically inner activated carbon block; it was used for both challenge testing and by FCS in the deployed AHS units. After filtration, water passes through a PearlAqua Micro 12C LED UV disinfection unit that delivers >16 mJ/cm^2^ fluence at the flow rates used, which provides supplemental treatment of already safe public water to reduce nonpathogenic bacteria. Flow in the AHS units is chilled after disinfection and prior to delivery via either a bubbler or a bottle-filler. The AHS unit is shown schematically in Figure 1.

**Figure 1:**
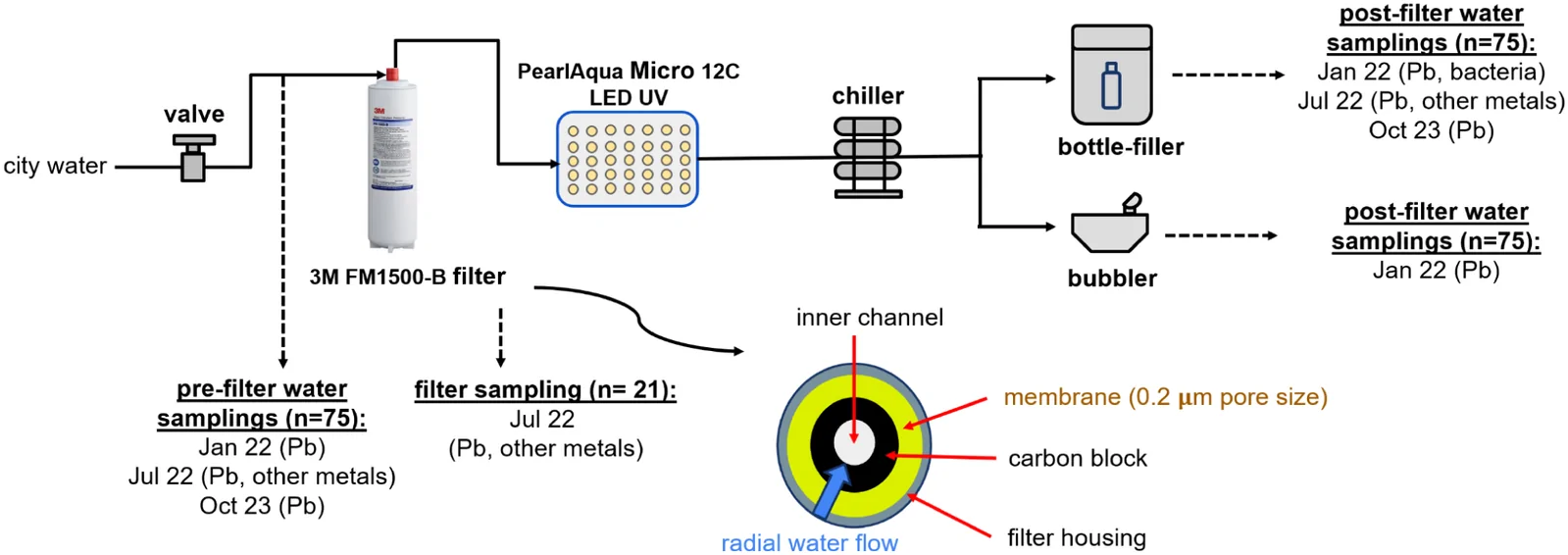
Overview of deployed AHS units design and field samplings.

### 2.2 Laboratory Challenge Testing

Before installing AHS units in the field, laboratory-based challenge testing was performed at Kettering University in Flint, MI using two AHS units that were the same as those deployed in the schools. Both lead/copper and bacterial challenge tests were included.

#### 2.2.1 Lead and Copper Challenge

Three rounds of metal challenge testing (in duplicate) were conducted to evaluate how well the AHS units remove lead and copper. Lead and copper challenge tests followed “lead pH 8.5 reduction test water” protocols in NSF Standard 53–2015 (section 7.4.3.5.2.3)^53^ (Table S1) except that a smaller volume of challenge water (20 L rather than 5678 L, which is the recommended capacity by the filter manufacturer) was used. Metal challenge waters were prepared using reverse osmosis (RO) water spiked at 150 to 170 μg/L lead using lead nitrate and 740 to 880 μg/L copper using copper­(II) sulfate pentahydrate. For each round, 10 L of distilled water was run as a prerinse, followed by 20 L of the test water, and finally 10 L of a postrinse. The water pressure was maintained between 25 and 40 psi, lower than recommended by the NSF standard but comparable to levels measured in FCS school buildings. The challenge testing unit setup was not conducive to the cyclic feed patterns recommended in NSF Standard 53 given limitations at the testing facility in Flint. Instead, the AHS units received a relatively constant flow rate between 25 and 56 L per h (lph), which required less than an hour to process all the feedwater. Water samples were collected from the influent and bottle filler at the end of the test phase for each round and were sent to a certified laboratory (Brighton Analytical, Brighton, MI) for total lead and copper analysis (EPA methods 200.8, rev 5.4).^54^ The analytical laboratory reports a detection limit of 1 μg/L for lead and 20 μg/L for copper.

#### 2.2.2 Bacterial Challenge Testing

Bacterial challenge testing was performed on three separate days under each condition using two distinct hydration stations. General Test Water #1 and Turbid Test Water #4, as defined by NSF Protocol P231, were used for bacterial testing.^55^ The Turbid Test Water #4 was included to evaluate the impact of particulate matter on bacterial cfu reduction by the membrane filter and LED UV–C components in the AHS units. Testing was conducted with *Raoultella terrigena* (ATCC 33257) using both Test Water #1 and #4, and *Pseudomonas aeruginosa* (ATCC 27853) using Test Water #1. *R. terrigena* is a coliform bacteria recommended by NSF Protocol P231. *P. aeruginosa* is a noncoliform biofilm-forming species detected in water collected from houses in Flint during the water crisis.^56^ The protocol for Test Water #4 indicates that parahydroxybenzoic acid should be added to just below the trigger point of the warning alarm on the UV unit to depress a water’s transmittance and allow testing under conditions where the water simulates reduced clarity. It was added at 5 mg/mL during the first run but was removed for runs 2 and 3; in all three runs, the Test Water #4 contained A2 Fine Dust to create a base level of turbidity >30 ntu for all runs. To condition the bacteria under low nutrient conditions before applying them to the AHS units, each strain was grown at 30 °C in serially diluted R2A media (1:250 R2A for *R. terrigena* and 1:90 R2A for *P. aeruginosa*).

The water pressure and flow rate was comparable to those in metal challenge tests except for one test with Test Water #4 that flowed at 6.4 lph due to a partial clog from the turbid water. Challenge tests were initiated by pumping prerinsewater (10 L), then pumping Test Water #1 or Test Water #4 (20 L) spiked with 1.3 × 10^5^ to 6.6 × 10^5^ cfu/ml of *R. terrigena* or 3.3 × 10^5^ to 8.0 × 10^5^ cfu/ml of *P. aeruginosa*. After each challenge test, AHS units were postrinsed by pumping 10-L distilled water. Water samples were taken from the influent and bottle filler (treated water) at the end of the test phase (before the postrinse phase). Total cells were measured using the heterotrophic plate counting (HPC) procedure (Standard Methods 9215) for *R. terrigena* and using Pseudomonas Isolation Agar (Difco) for *P. aeruginosa*. All cell count culture plates were incubated at 30 °C.

### 2.3 Field Performance Testing for Lead, Bacteria, and Other Metals

Three field sampling events occurred: January 2022, July 2022, and October 2023 (Figure 1). FCS buildings were shut down over two months due to pandemic-related outbreaks and a holiday break prior to the January 2022 sampling. This sampling was conducted by Arch Environmental (Detroit, MI). The July 2022 sampling occurred after a low usage time in school buildings for about one month and prior to the start of a new school year. The October 2023 sampling was the only sampling event during a school period when AHS units and water lines were actively being used in buildings. Consequently, the three sampling events represented different building and HyS use patterns.

During all three field campaigns, prefilter and corresponding post-AHS water samples were collected based on protocols recommended by EPA 3Ts.^57^ A first draw sample (∼250 mL) was taken after overnight stagnation. While EPA 3Ts recommends sampling a total of 5 outlets in each school to provide an overall understanding of the prevalence and spatial variation of lead in schools, we only sampled before the filters and the AHS-treated water to assess the performance of AHS under field conditions. Plumbers accompanied the research team to provide prefilter sampling access and selected the order of school buildings and AHS units to be sampled. Prefilter samples were obtained by shutting the wall isolation valve off, removing the tubing line at the filter inlet, and then slowly opening the wall isolation valve for controlled sample collection from the inlet tubings. Post-AHS samples refer to those taken at the bottle fillers and/or bubblers. For the January 2022 sampling, water samples were collected at each hydration station from the bubbler first, then bottle filler, then from the supply line (prefilter). This order was reversed for the July 2022 and October 2023 samplings with no bubbler samples collected because they had been turned off due to pandemic-related concerns. A list of sampling locations in different school districts is provided by Table S5. All samples for total lead analysis were acidified (1% w/w) using trace metal-grade nitric acid either in the field or at the analytical lab the same day as samples were collected. These samples were then analyzed by Brighton Analytical (Brighton, MI) using EPA method 200.8, rev 5.4. Five sequential 250 mL prefilter water samples were collected during the October 2023 sampling period in eight locations where at least one grab sample during prior sampling events had lead concentration above or close to 100 μg/L. Out of an abundance of caution due to the overall high lead level in school building A, sequential samples were taken for all four locations despite the fact that A1–2 and A1–4 had relatively lower lead levels detected. Microbial monitoring was conducted on bottle-filler samples from January 2022 using sterile bottles. Samples were analyzed for heterotrophic plate count (HPC) bacteria (Standard Methods protocol 9215B), total coliforms and total *Escherichia coli* (Standard Methods protocol 9223B), also by Brighton Analytical.

During the July 2022 sampling, the AHS filter was removed and replaced with a new filter (labeled with installation date) after all water sampling was complete. Used filters were emptied of residual water before being stored in individual 2-gallon Ziploc bags. Filters were eventually double-bagged, placed in shipping buckets, and mailed to Washington University in St. Louis, Missouri for further testing. Ultimately, 21 of these filters were used for total lead extraction analysis. Additionally, both the prefilter and postfilter water samples (July 2022) associated with these 21 filters were collected from the analytical lab after lead analysis was complete and taken to Wayne State University for analysis of metals other than lead (Section S3).

Local information about the condition of each AHS and the usage indicator (# of bottles saved) were recorded for the July 2022 sampling. The number of bottles saved (volume in L assuming 0.59 L per bottle) prior to changing the filter ranged from 34 (20.1) to 75,694 (44,659) with an average of 3,118 (1840) and median of 1,229 (725) (n = 67 recorded with the remaining 4 sampled AHS units not providing # of bottles saved information). The average and median were within the manufacturer’s recommended capacity of the filter (5,678 L) and only 3 AHS units exceeded it. Note that bottle saving counters were not reset for all AHS units when new filters were installed after July 2022; therefore, information on bottles saved is not reliable for the October 2023 sampling and was not used.

### 2.4 Field Filter Lead Extraction Analysis

#### 2.4.1 Lead Extraction from Field Filters

##### 2.4.1.1 Overall Approach

Lead was extracted from 21 used NSF53-certified POU filters harvested in July 2022 to estimate the total mass of lead that had accumulated on the filters. These 21 filters were selected as those with the highest known cumulative volumes and those with the highest influent lead concentrations determined from grab samples. These extractions provide estimates of the total mass of lead in the water that had entered the filters and then accumulated on the filter media. Extractions were performed by passing at least 7 L of 1 M HNO_3_ (ACS, 68.0–70.0%, purchased from Thermo Scientific) through the POU filters at a flow rate of 20 mL/min; most extractions used 7 L, but a few used 10 L. For extractions using 7 L of extractant, the first 2 L of effluent were collected in 0.5-L increments and the remaining 5 L were collected in 1-L increments (Figure S3). For extractions performed with 10 L of extractant, all effluent samples were collected in 0.5-L increments. Each extraction increment was analyzed by inductively coupled plasma mass spectrometry (ICP-MS) to quantify the total concentration of lead. The mass of lead extracted in each extract volume (m_i_) was calculated by multiplying the concentration of lead (C_i_) by the volume of extract solution (V_i_) (eq 1). The total mass of lead extracted from a filter (m_extracted_) was calculated as the sum of the mass in each extract volume analyzed (eq 2).

1 $$m_{i}=C_{i}\times V_{i}$$

2 $$m_{\mathrm{extracted}}=\sum \mathrm{mi}$$

There are two fundamental assumptions underlying this approach. The first is that NSF53-certified POU filters are able to capture all lead in the water entering them. The second is that all of the accumulated lead can be extracted with the passage of 7 L of 1 M HNO_3_ at 20 mL/min. We confirmed that the first assumption is valid in tests in which influent waters with known amounts of lead were fed into the filters and the effluent waters were measured (Figure S1). It is also consistent with the certification of the filter for the removal of lead, the laboratory challenge tests done at Kettering University, and our analysis of grab water samples taken during field campaigns (Section 3.2.1).^51^ It has also been reported in other field studies that NSF53-certified POU filters remove almost all incoming lead.^32,34,35,50,58^

We assessed the validity of the second assumption in laboratory-based tests using filters with known amounts of accumulated lead. Extraction using 6–7 L of 1 M HNO_3_ at a flow rate of 20 mL/min achieved almost complete recovery (98.8% ± 8.9%) of lead that had accumulated on the filters, which were loaded using influent solutions with dissolved lead (Table S4 and Figure S2). The complete extractability of lead from the filters is consistent with previous work using 1 M HNO_3_ to extract dissolved lead accumulated on faucet-mounted filters,^48,50^ which also contain solid block activated carbon. In addition to extracting lead that had accumulated on the filters from the introduction of dissolved lead, this approach can also fully extract particulate lead from faucet-mounted filters^49,50^ and is likely to be able to extract particulate lead from the filters in the hydration stations.

##### 2.4.1.2 Volume-Averaged Concentrations

The volume-averaged concentration (*C*_*avg*_) is the average concentration of lead in upstream water throughout the duration of the filter’s use. *C*_*avg*_ in the water entering the filters was calculated by dividing the measured total mass of lead extracted from the filter (*M*_*extracted*_) by the cumulative volume of water that had run through a filter before it was harvested (*V*_*cum*_) (eq 3). Cumulative volume information was calculated using information on “# of bottles saved” reported by the hydration station through a conversion unit of 0.59 L per bottle.^59^

3 $$C_{\mathrm{avg}}=\frac{M_{extracted}}{V_{cum}}$$

This approach for calculating volume-averaged concentrations was applied to other metals (copper, aluminum, iron, zinc and manganese). However, the two above-mentioned assumptions have not been verified in lab-based tests for these metals. For the first assumption, it was observed from field grab samples that these metals were not always completely removed by POU filters as will be discussed in Section 3.2.3. While we have not verified the second assumption, which is that these metals will be fully extracted, it is likely that they will be because they have high solubility and low adsorption tendency at low pH (pH ≈ 0 for 1 M HNO_3_) and hence a high tendency to be dissociated from the activated carbon media of the POU filter.^60–64^ While there are limitations on the quantitative accuracy of this approach for these metals, valuable information on their collective accumulation on AHS can still be provided.

#### 2.4.2 Metals Analytical Methods

Extract samples that contained Pb, Cu, Fe, Al, Zn, and Mn were diluted and acidified to 1% w/w acid solution using ultrapure water (>18.2 mΩ-cm) and trace metal-grade nitric acid (Fisher Scientific). Samples were then analyzed via ICP-MS (NexION 2000 PerkinElmer) in the Chemical and Environmental Analysis Facility at Washington University in St. Louis following EPA Method 200.8, rev 5.4. Analysis of Cu, Fe, Al, Zn, Mn and Ni in influent and effluent water samples (July 2022) associated with these filters was detailed in Section S3.

## 3 Results and Discussion

### 3.1 Challenge Testing Supported Field Installation of AHS Units

#### 3.1.1 Lead and Copper Removal

AHS units effectively removed lead and copper in lab-based challenge tests. For all six experiments, lead was removed to below the detection limit (1 μg/L) (Table S2). For the first five of six tests, copper was removed to below the detection limit (20 μg/L), and in the sixth test, copper was removed to 20 μg/L. This result met the expectations of FCS administration for lead and copper water quality in AHS treated drinking water and supported the field installation of AHS units.

#### 3.1.2 Bacteria Removal

The AHS units were shown to be capable of effectively removing bacteria during laboratory challenge testing (Table S3), although the mechanism of removal was not explicitly elucidated. *R. terrigena* (surrogate coliform bacteria) was removed from Test Water #1 at log reduction values (LRV) > 2.6 in one test run for each experimental AHS and >3.4 for the remaining two test runs in both experimental AHS units. Throughout all tests, only one colony forming unit was detected (run #3, Unit #2). Similarly, in Test Water #1, *P. aeruginosa* (noncoliform, biofilm-forming bacteria) was removed at LRVs > 3.9, with no colonies detected on three of the six AHS experiments. Finally, in turbid Test Water #4, *R. terrigena* was removed at a LRV > 4.1 with no colonies detected across all runs. This result met the expectations of FCS administration for bacterial water quality in AHS treated drinking water.

### 3.2 Field-Deployed AHS Units Improved Water Quality in Filtered Water

#### 3.2.1 Single Grab Lead Samples

Filters installed in AHS units were robust lead removal treatment units. For all three sampling events (January 2022, July 2022, and October 2023) when a filter had been used for six months or longer, the AHS units had routinely removed total lead so that lead concentrations in the water delivered by the AHS were below detection (1 μg/L) in all but a few units (98% of units, Table S5, Figure 2). The influent water to hydration stations sampled had median total lead concentrations of 12 μg/L, 6 μg/L, 14 μg/L and maximum total lead concentrations of 620 μg/L, 190 μg/L, 470 μg/L for these three sampling campaigns, respectively. About 53% of influent water samples had total lead concentrations above 10 μg/L. Despite these high lead loads entering the AHS units, only two units in July 2022 and two in October 2023 had detectable but low (<3 μg/L) lead levels in treated water samples. Note that we had reliable field data on the volume filtered through the AHS units for the July 2022 sampling. All but three of the units for which the # of bottles saved was recorded (n = 67 out of 71 sampled) had total filtered volumes less than the manufacturer's recommended volume limit.

**Figure 2:**
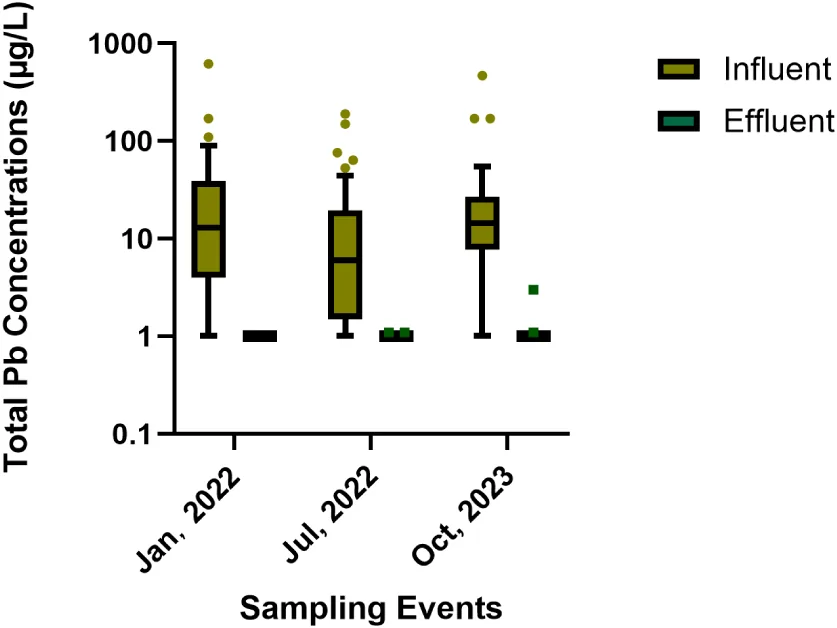
Lead in influent and effluent grab samples for hydration stations collected in January 2022 (*n* = 74), July 2022 (*n* = 71) and October 2023 (*n* = 60). These are Tukey graphs that indicate the minimum, first quartile, the median, third quartile, and the maximum values. Outliers are shown in individual dots. For samples that had total lead concentrations below the detection limit, the detection limit of 1 μg/L was used to represent the concentration in the figure.

Total lead entering most AHS units varied over the three sampling events (Section S4). Overall, the majority of AHS units exhibited highly varied levels of lead concentrations in the influent grab samples with an average coefficient of variation of 82% (Figure S7­(d)). This suggests that lead concentrations in the influents to the AHS units are not consistent over time and are strongly time-variant. This result also highlights the value of volume-integrated composite lead samples to provide information on overall avoided lead exposure. Variations in stagnation patterns before samples were taken probably contribute to this temporal fluctuation.

#### 3.2.2 Sequential Lead Samples

Sequential sampling at eight selected units that routinely had high lead levels entering AHS units indicates that the main lead sources are likely to be spatially close to the AHS units and may be associated with isolation valves installed immediately upstream of the units. For AHS units that had high incoming lead levels in the January 2022 and July 2022 sampling events (Section S4), a sequential sampling series with five 0.25-L increments (1.25-L total) was performed in October 2023. Average total lead concentrations showed an overall decreasing trend over sequential samples (Figure 3). The first 0.25-L samples usually, but not always, had the highest lead concentrations with an average of 95.0 μg/L total lead, while the fifth 0.25-L samples had an average of 6.2 μg/L. This suggests that lead-containing isolation valves are a likely lead source. However, because there was still detectable lead in the fifth samples for some AHS units, more upstream plumbing components may also be releasing lead into the water. Individual total lead concentrations in sequential samples for all eight AHS units are included in Section S5.

**Figure 3:**
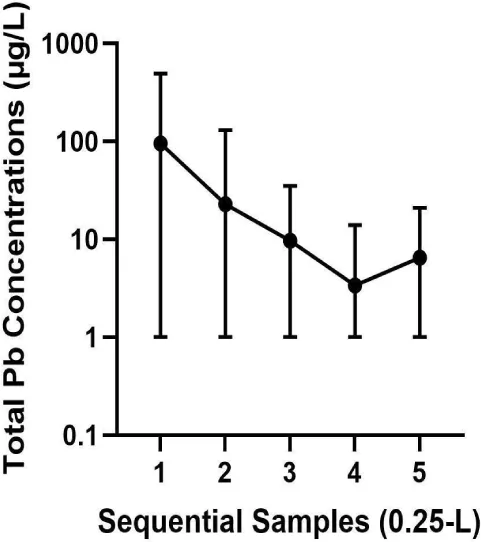
Sequential sampling series taken in October 2023 for eight AHS units that had high lead concentrations in January 2022 and July 2022 influent water samples. For samples that had undetectable lead levels, the detection limit of 1 μg/L was entered as the total lead concentration. Dots represent the median total lead concentrations among eight locations for each sequential sample. The variations represent the range of total lead concentrations taken at all eight locations for each sequential sample.

#### 3.2.3 Metals Other Than Lead

Metals other than lead were removed by HyS units to varying levels (Section S3). Zinc, aluminum and manganese were substantially lower in the effluent than in the influent, while the extents of removal of copper, iron and nickel were measurable but not as great as those for zinc, aluminum, and manganese. Some HyS units contained higher levels of metals in the effluent water samples than in the influent water samples. This could be a result of release from postfiltration plumbing under interaction with filtered water, which can be more corrosive.^33,65^ Detailed discussions are included in Section S3.

#### 3.2.4 Bacterial Analysis

The field-deployed AHS units that included membrane filters and LED UV–C lamps were found during the January 2022 sampling event to be effective at removing HPC bacteria. No bacterial colonies were detected in any water sample collected from the AHS bottle filling spigots but were very likely to be present in influent water.^66–70^ The LED UV–C lamps used have a fluence range >16 mJ/cm^2^ at the flow rates experienced, which is effective for HPC bacteria inactivation (LRVs > 3) and consistent with our inability to detect HPC colonies from filtered field water samples.^71^ The cell reduction in the field was undoubtedly also substantially, if not mostly, supported by the microporous membrane that preceded the LED UV–C unit. Between this field result and the lack of detectable bacteria during the laboratory challenge tests, we discontinued bacterial monitoring during subsequent sampling campaigns. The results demonstrate that the AHS units helped to reduce bacterial counts that have otherwise been shown across multiple studies to increase in filtered water.^37,39–41,56^ Because bacterial samples were not collected between the filter and LED UV–C lamp in monitored units, we cannot estimate the relative contribution of the membrane filter and the LED UV–C lamp to bacterial removal.

### 3.3 Filters Served as Volume-Integrated Lead Monitoring Devices

#### 3.3.1 Volume-Averaged Lead Concentrations

Volume-averaged lead concentrations in the water entering these 21 hydration stations ranged from below 0.1 μg/L to 13.7 μg/L with a median of 0.8 μg/L (Figure 4). Among them, there were eight units that had volume-averaged lead concentrations over 1.0 μg/L. Because these are volume-averaged concentrations, the lead levels in the water flowing into a given hydration station would have sometimes been below and sometimes above the volume-averaged values. Lead concentrations in water depend on the water use patterns, such as the duration of stagnation prior to use and the composition of plumbing materials in which the water had stagnated.^15,16,27,72,73^ Consequently, the concentrations of lead coming into a hydration station or other fixture can be highly temporally variable, as observed in grab samples (Section S4). The concentration of a grab sample only provides information on that exact parcel of water, but the volume-averaged concentration gives overall information on the average lead loading to a hydration station. Extraction profiles of all filters are included in Section S6.

**Figure 4:**
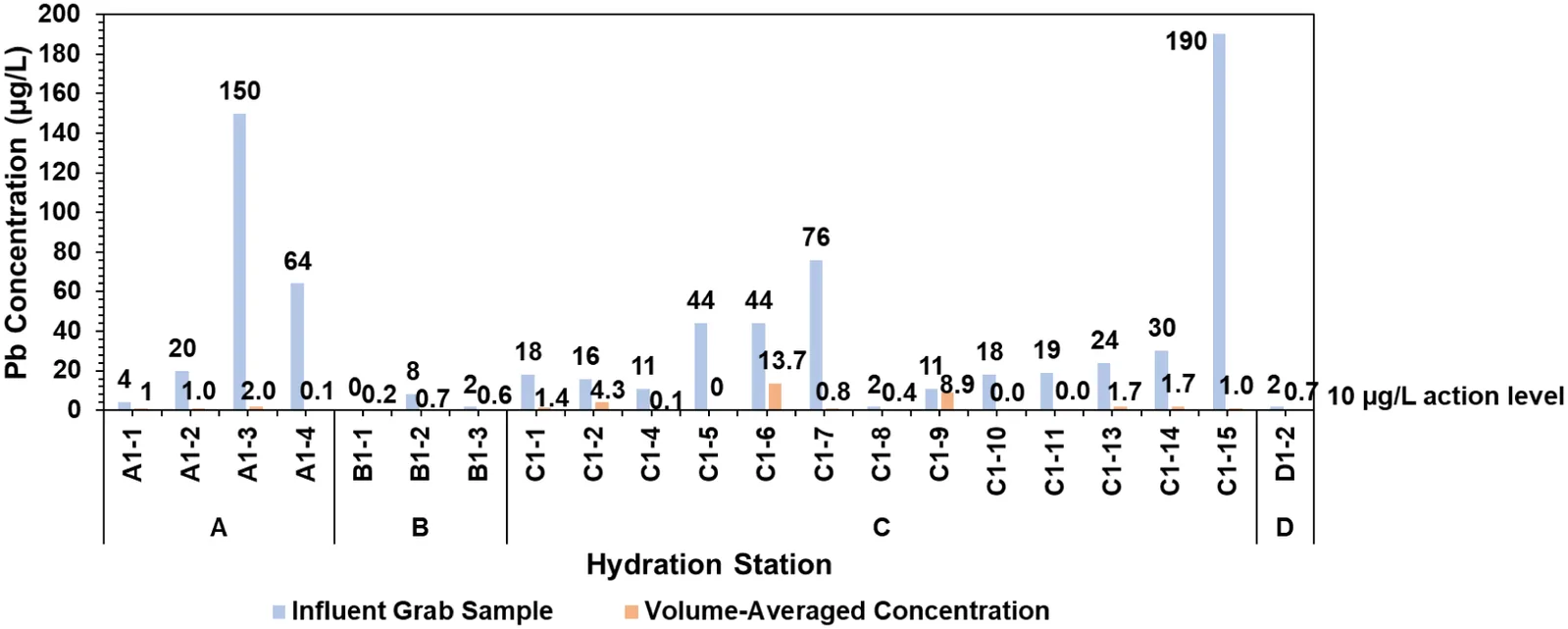
Single grab concentrations versus volume-averaged Pb concentrations in water entering hydration stations (*n* = 21). Each label on the *x*-axis represents one hydration station. The first letters represent the name of the building where the hydration station was located. The number before the hyphen represents the floor level where the hydration station was located. The number after the hyphen refers to an individual hydration station on a certain floor in a certain building. Hydration station filters that were acid-extracted experienced a wide range of processed water volume (136 L to 44,700 L).

#### 3.3.2 Influent Grab Sample Lead Concentrations

For almost all 21 hydration stations analyzed, the lead concentrations in the prefilter grab samples were higher than the volume-averaged concentrations (Figure 4). B1–1 had low grab sample concentration (below detection limit, 1 μg/L) and volume-averaged concentration (0.2 μg/L). Most (15 of 21) grab samples had lead concentrations greater than 10 μg/L, while all except one volume-averaged concentrations were below this level. These prefilter grab samples were taken in the morning in July 2022 when schools were not in session. Their relatively high lead concentrations are consistent with the long stagnation times expected for this scenario.^15,16,33^

Influent grab sample lead concentrations and volume-averaged lead concentrations for hydration stations provide complementary information that can be employed together to assess lead sources in the upstream plumbing (Figure 5). For hydration stations that have both low influent grab concentrations (<10 μg/L, new trigger level) and low volume-averaged concentrations (<1 μg/L, 1 order of magnitude lower than the new trigger level), it is likely that there is not a substantial source of lead upstream (6 out of 21 hydration stations, green). For hydration stations with both high volume-averaged concentrations and high influent grab concentrations, there are almost certainly substantial sources of lead upstream (7 out of 21 hydration stations, red). These hydration station locations should be prioritized for replacement of upstream plumbing.^4^ These sources may be lead-containing fixtures, solders, or brass fittings.^74–78^ Hydration stations that have high influent grab sample concentrations but low volume-averaged concentrations may be those for which lead release is most susceptible to long stagnation time (8 out of 21 hydration stations, yellow); for these hydration stations, a daily flushing protocol after overnight stagnation could be effective in lowering lead concentrations.^79^ No hydration stations had high volume-averaged concentrations but low influent grab concentrations. Volume-averaged concentrations calculated from acid extraction can be used to quantify avoided lead exposure and identify potential hot spots of lead release within old buildings that might not be identified using grab samples. For example, the hydration station circled in red may not be prioritized for lead control actions based solely on grab samples. However, it had the second highest volume-averaged concentration (8.9 μg/L), which suggests that it would pose one of the highest risks of lead exposure through drinking water if the AHS had not been installed.

**Figure 5:**
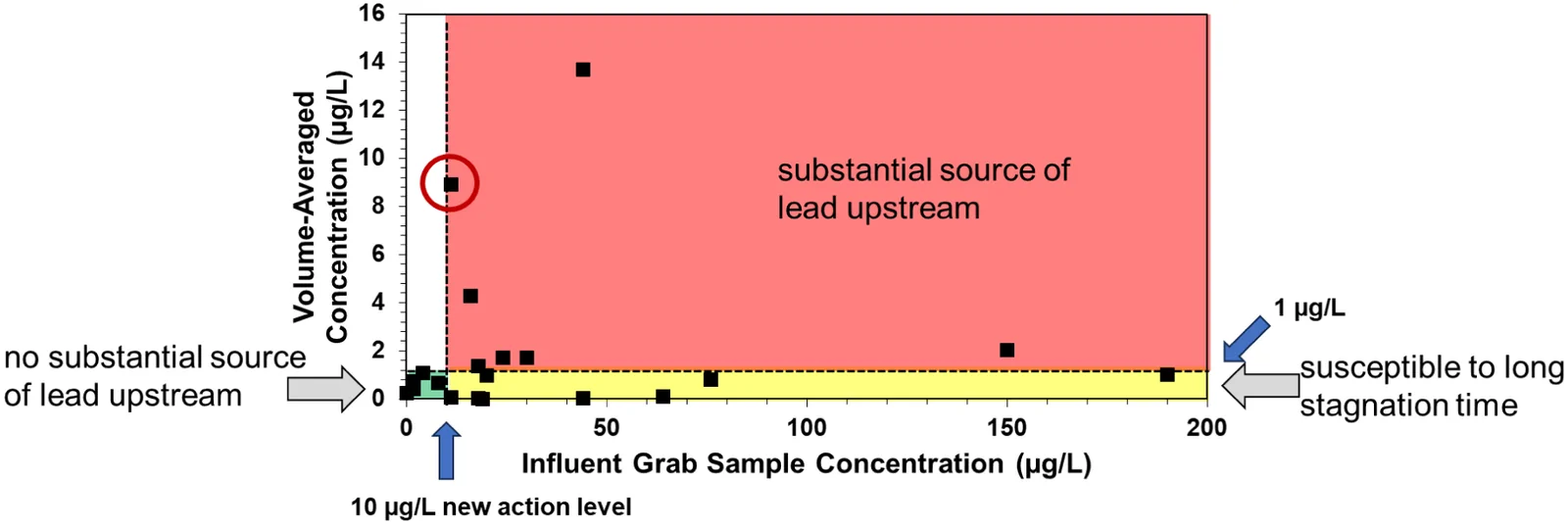
Quadrant model of hydration stations with different influent grab sample lead concentration and volume-averaged lead concentration scenarios.

#### 3.3.3 Metals Other Than Lead

Volume-averaged and influent grab sample copper, iron, aluminum, zinc and manganese concentrations in the upstream water of each hydration station analyzed are shown in Section S7. No hydration station experienced concerning levels of these metals. There are no significant linear relationships between the pairs of total metal masses extracted (Section S8). There are also no significant linear relationships between the total mass of a metal extracted and the cumulative volume as all R^2^ are below 0.1 (Section S9). This result suggests that variability in water use patterns, especially stagnation durations prior to use, water chemistry and the exact composition of upstream distribution system and premise plumbing materials are probably the primary causes of variations in the metal concentrations in the influent water to different hydration stations.

## 4 Environmental Implications

In this study, we demonstrated that AHS units in schools are robust treatment units for removing lead. Our lab-based challenge tests and field samples both confirmed that the solid block activated carbon inside HyS units effectively removed incoming lead to yield very low concentrations (usually below the detection limit) in the filtered water. As use of POU filtration systems continues to grow across the country in response to concerns about lead in drinking water and associated Filter First legislation, our work confirms the effective lead removal by field-deployed HyS units that use NSF-certified filters. While our study explored the effectiveness of bacterial HPC count reduction during one sampling event, more work is encouraged to ascertain the relative contribution of activated carbon block filters, membrane filters, and LED–UV-C treatment in HyS units to produce low HPC drinking water. Future work on the extent of virus removal by filtration and UV–C systems is also encouraged.^80,81^

We validated a method to recover accumulated lead from school filters and illustrated the value of the volume-averaged lead concentrations obtained from this approach. Volume-averaged lead concentrations provide quantitative insight into avoided lead exposure, whereas current grab sampling provides information about a single parcel of water at the time of sampling. This filter-based lead monitoring approach can be effective at assessing upstream sources of lead, identifying priority locations for lead mitigation, and quantifying the benefits of using the filters. To implement our filter-based lead monitoring approach, we recommend schools to prioritize outlets where grab samples indicate high lead concentrations. Future work can focus on extractions for other metal contaminants, modeling avoided metals exposure risks and calculating associated health benefits.

## Supplementary Material



## Notes

Laboratory challenge testing; lead extraction protocols and validations; metals other than lead in grab samples; total lead and its variations in influent grab samples; sequential total lead concentrations; lead extraction profiles of field-deployed school filters; volume-averaged concentrations of metals other than lead; correlation between pairs of total extracted metals; and correlation between total extracted metals and cumulative volume (PDF)
