# Supplementary material for: Monitoring Advanced Hydration Station (AHS) Water Quality in Flint, MI, School Buildings: Field Grab Sampling and Filter-Based Lead Assessment
Source: Environ Sci Technol. 2026 Oct 23;60(44):32069–78. doi: 10.1021/acs.est.6c09190 (PMC13757953; doi:10.1021/acs.est.6c09190)
Supplement: Supplementary file 1 [file es6c09190_si_001.pdf]

Supporting Information

Monitoring Advanced Hydration Station (AHS) Water Quality in Flint, MI School Buildings: Field Grab Sampling and Filter-based Lead Assessment

Zehua Wang[1a], Laura Sullivan[2a], Michelle Ammerman[3], Jean M. Brownell[1], Anushka Mishrra[1], Conor Gowan[4], Shawn P. McElmurry[4], Daniel E. Giammar[1], Nancy G. Love[3*]

1. Department of Energy, Environmental and Chemical Engineering, Washington University in St. Louis, St. Louis, Missouri 63150, United States
2. Department of Mechanical Engineering, Kettering University, Flint, Michigan 48504, United States
3. Department of Civil and Environmental Engineering, University of Michigan Ann Arbor, Ann Arbor, Michigan 48109, United States
4. Department of Civil and Environmental Engineering, Wayne State University, Detroit, Michigan 48202, United States

*corresponding author: nglove@umich.edu, phone: (734) 763-9664
[a]co-first authors: these two authors contributed equally to this work

This file contains 24 pages, including 12 figures and 5 tables.

submitted to *Environmental Science & Technology*

Table of contents

Section S1. Laboratory lead/copper and bacteria challenge testing. S3

Section S2. Assessment of lead recovery effectiveness from lab-loaded filters and extraction procedure of field-deployed school filters. S6

Section S3. Metals other than lead in grab water samples (July 2022). S8

Section S4. Total lead in influent grab samples taken across three field samplings. S10

Section S5. Individual total lead concentrations in sequential samples for all eight AHS units. S15

Section S6: Lead extraction profiles of POU filters removed from hydration stations. S16

Section S7. Single grab and volume-averaged concentrations of metals other than lead. S20

Section S8. Correlation between masses of total extracted metal. S21

Section S9. Total metals extracted versus cumulative water flow through filters. S24

Section S1. Laboratory lead/copper and bacteria challenge testing.

Table S1. Lead/copper challenge water was prepared in accordance with NSF_53 2015 section 7.4.3.5.2.3.2 using RO water in accordance with instructions, producing a feed solution with the characteristics shown in the table.

Species	Value	Unit
pH	8.5	/
alkalinity	100	mg/L as $CaCO_3$
hardness	100	mg/L as $CaCO_3$
total chlorine	0.5	mg/L
Ca^{2+}	27.2	mg/L
Mg^{2+}	8.2	mg/L
SO_4^{2-}	34.0	mg/L
Na^{+}	45.4	mg/L
Cl^{-}	48.3	mg/L
NO_3^{-}	34.2	µg/L
Pb (total)	150	µg/L
Cu^{2+}	750	µg/L

Table S2. Lead and copper challenge test results

		Unit #1			Unit #2		
**Run #**		**PSI, gal/hour**	**Input Conc. (µg/L)**	**Output Conc. (µg/L)**	**PSI, gal/hour**	**Input Conc. (µg/L)**	**Output Conc. (µg/L)**
1	lead	37, 13.2	170	N.D.	36, 14.8	170	N.D.
	copper		820	N.D.		880	N.D.
2	lead	37, 13.2	150	N.D.	36, 14.8	150	N.D.
	copper		750	N.D.		750	20
3	lead	35, 13.6	150	N.D.	36, 14.8	150	N.D.
	copper		740	N.D.		740	N.D.

Note: ‘N.D.’ represents not detected. The reported limits of lead and copper method quantification are 1 µg/L and 20 µg/L, respectively.

Table S3. Bacterial challenge test results

***Raoutella terrigena*, Test Water #1**						
	**Unit #1**			**Unit #2**		
**Run #**	**PSI, gal/hour**	**cfu/mL applied (cfu/mL out)**	**LRV**	**PSI, gal/hour**	**cfu/mL applied (cfu/mL out)**	**LRV**
1	25-32, 14.1	2.0×10^5 (0)	>2.6	36, 13.2	2.6×10^5 (0)	>2.7
2	38-40, 6.7	1.3×10^5 (0)	>3.4	35, 12.7	1.6×10^5 (0)	>3.5
3	32-36, 9.4	3.0×10^5 (0)	>4.5	34, 12.4	3.0×10^5 (1.0×10^1)[a]	4.5
***Pseudomonas aeruginosa*, Test Water #1**						
1	36, 9.0	7.9×10^5 (9.0×10^1)[a]	3.9	36, 12.4	3.3×10^5 (1.0×10^1)[a]	4.5
2	34, 10.9	7.0×10^5 (2.0×10^1)[a]	4.5	36, 11.3	4.7×10^5 (0)	>4.7
3	33, 11.3	4.7×10^5 (0)	>4.7[a]	36, 11.6	7.4×10^5 (0)	>4.9
***Raoutella terrigena*, Test Water #4**						
1	40, 1.7	6.6×10^5 (0)	>4.8	36, 7.8	5.6×10^5 (0)	>4.7
2	34, 13.2	2.1×10^5 (0)	>4.3	34, 14.9	2.5×10^5 (0)	>4.4
3	34, 14.0	2.1×10^5 (0)	>4.3	34, 14.0	1.4×10^5 (0)	>4.1

LRVs (Log Reduction Values) were defined by dilutions used; [a]detection limit is 10 cfu/ml

Section S2. Assessment of lead recovery effectiveness from lab-loaded filters and extraction procedure of field-deployed school filters.

Lead challenge solution (60 L) was prepared using lead nitrate (Acros Organics) and ultrapure water (resistivity > 18.2 MΩ-cm) to yield a spiked lead concentration at 10 mg/L. This total mass of lead in the challenge solution was comparable to the level expected with the manufacturer's recommended volume. The spiked challenge solution was loaded onto a new NSF53-certified POU filter (FM DWS 1500-B) at a loading flow rate of 20 mL/min (**Figure S1**). The masses of lead loaded onto filters (m_{loaded}) were calculated by multiplying the concentration differences of influents ($C_{influent}$) and effluents ($C_{effluent}$) by the volume of loading solution ($V_{loading}$) (**Equation S1**). Acid extraction with 1 M nitric acid at an extraction flow rate of 20 mL/min was then conducted to recover loaded lead on filters. The masses of lead extracted by nitric acid were calculated as described in the main manuscript (**Equation 2**). Loading and subsequent extraction were performed using two replicate filters.

$$m_{loaded} = (C_{influent} - C_{effluent}) * V_{loading} \qquad \textbf{(Equation S1)}$$

Table S4. Amount of lead loaded onto and extracted from POU filters at a flow rate of 20 mL/min.

Mass in influent (mg)	Mass loaded (mg)	Mass extracted (mg)
591 ± 9	588 ± 10	581 ± 6

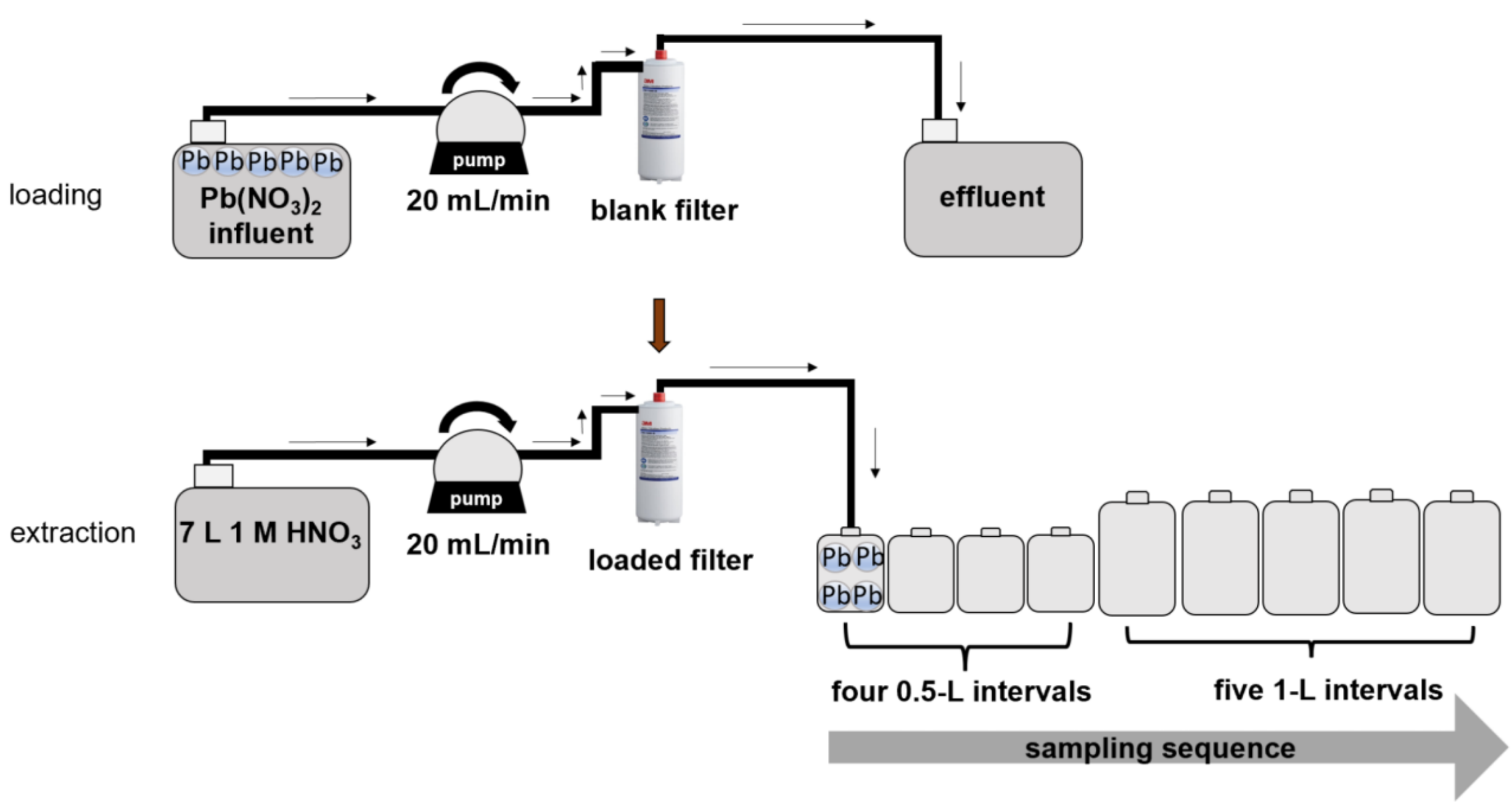

Figure S1. Loading dissolved lead in $Pb(NO_3)_2$ solution onto blank filters and subsequent extraction with 1 M HNO_3

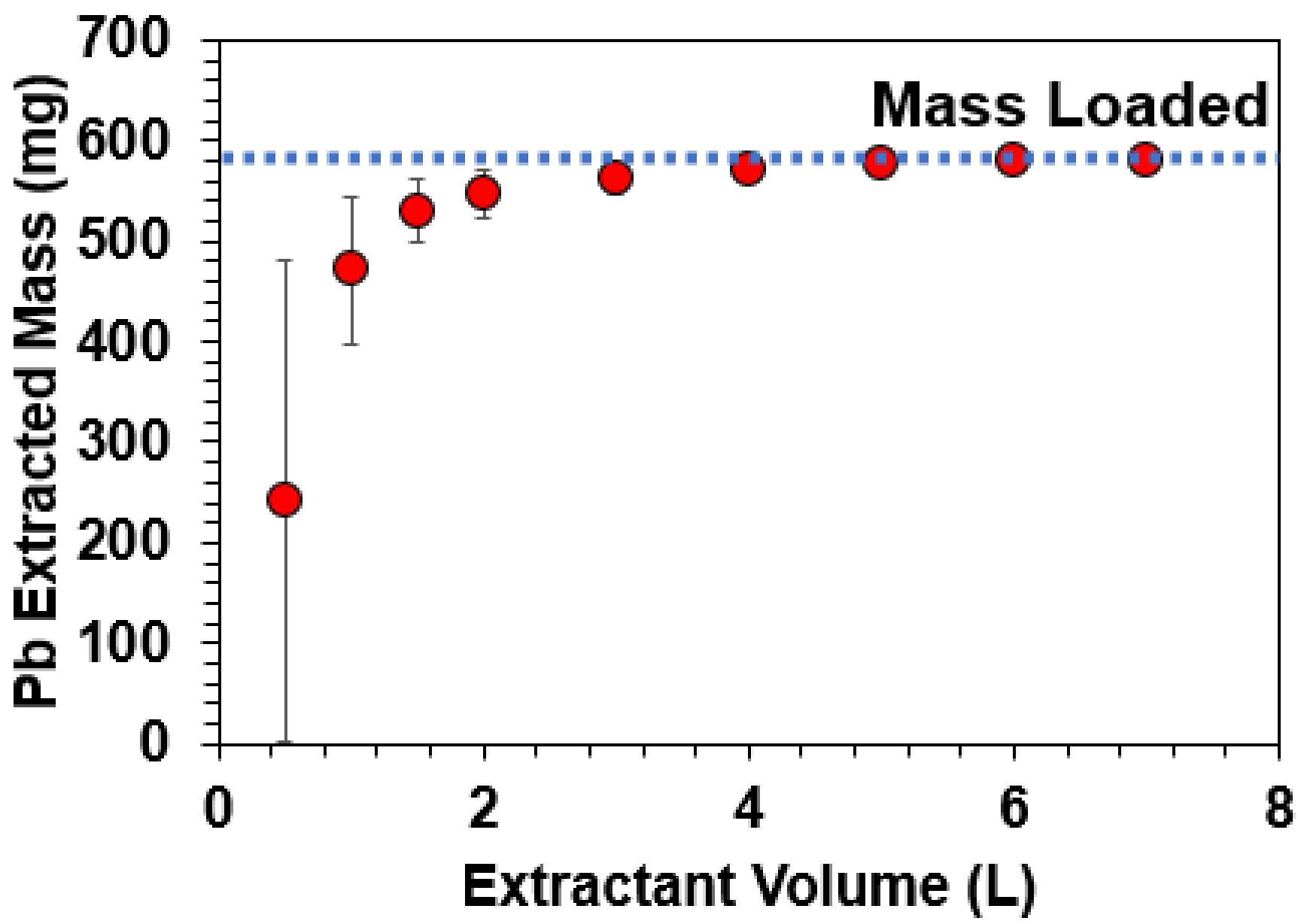

Figure S2. Cumulative lead extracted mass during extraction using 1 M nitric acid at 20 mL/min. Error bars represent variations from duplicate filters.

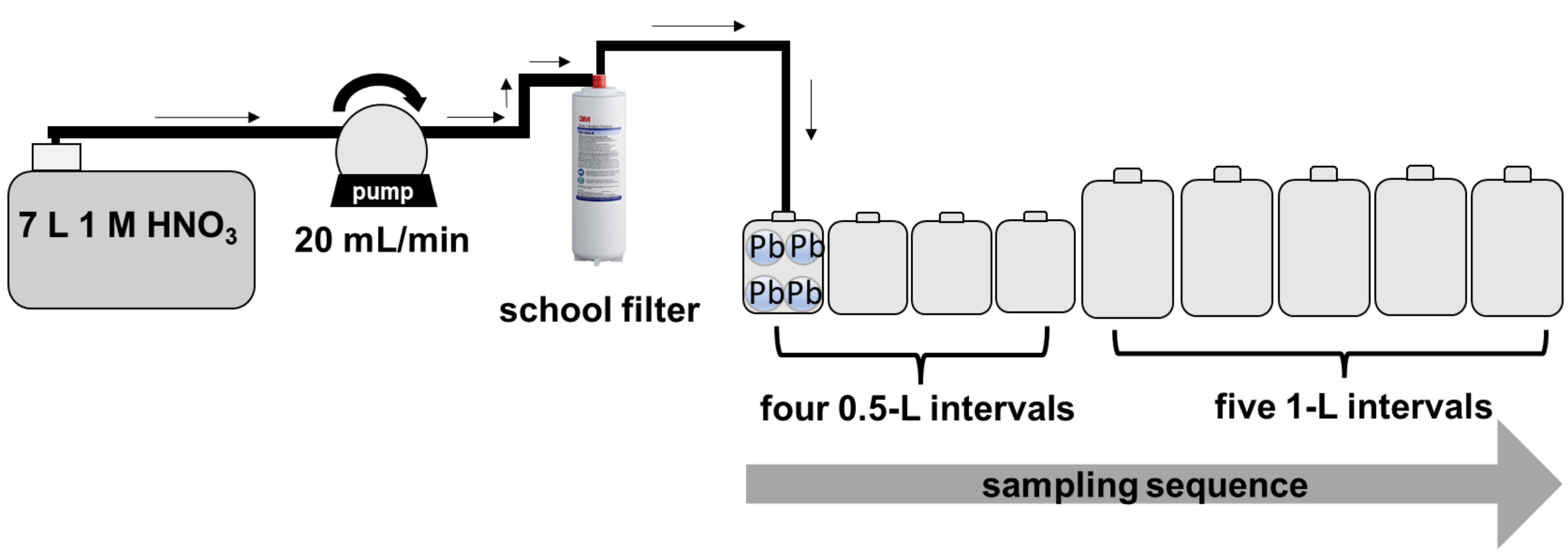

Figure S3. Acid extraction of used POU school filters with 1 M HNO_3 to quantify the masses of lead in the influent water to the filters that had accumulated on the filter media.

Section S3. Metals other than lead in grab water samples (July 2022).

Metals Analytical Methods: AHS influent and effluent grab samples (July 2022) associated with the 21 filters that were acid-extracted were acidified to 2 % w/w acid solution using ultrapure water (>18.2 mΩ-cm) and trace metal-grade nitric acid (Fisher Scientific). Samples were then analyzed via ICP-MS (Agilent 7700x) at the Lumigen Instrument Center at Wayne State University following EPA Method 200.8, rev 5.4 for Pb, Cu, Fe, Al, Zn, Mn and Ni with Bi (for Pb) and Sc (for Al, Mn, Fe, Ni, Cu and Zn) internal standards. Blank samples were below the limit of detection and check standards (200 µg/L and 30 µg/L) were within 15 % of expected concentrations.

Grab Water Samples Metals Analysis: For the 21 HyS units from which filters were extracted in July 2022, influent and effluent grab samples were analyzed for copper, iron, zinc, aluminum, manganese and nickel concentrations (**Figure S4**). For zinc, aluminum, and manganese, their mean influent concentrations were 505 µg/L, 576 µg/L, and 29 µg/L, respectively, and their mean effluent concentrations were 250 µg/L, 231 µg/L, and 8 µg/L, respectively. For copper, iron, and nickel, their mean influent concentrations were 654 µg/L, 315 µg/L, and 28 µg/L, respectively, and their mean effluent concentrations were 475 µg/L, 299 µg/L, and 23 µg/L, respectively. There were nine HyS units that had higher concentrations of copper in effluent samples than in influent samples, seven HyS units that had higher concentrations of iron or zinc in their effluent samples, four HyS units that had higher concentrations of aluminum, manganese or nickel. One HyS unit (D1-2) had elevated levels of copper, iron, zinc, aluminum, manganese and nickel in its filtered water.

Among the metals analyzed, two pairs showed significant linear correlations in influent grab-sample concentrations. Zinc and copper were correlated with an R^2 of 0.81, and lead and manganese were correlated with an R^2 of 0.91 **(Figure S5)**. Upstream brass (an alloy that contains copper and zinc) plumbing materials were probably vulnerable to prolonged stagnation prior to sample collection. These materials could be a common source of these two metals. Other pairs of metals in influent grab samples did not have significant linear correlation (**Figure S6**).

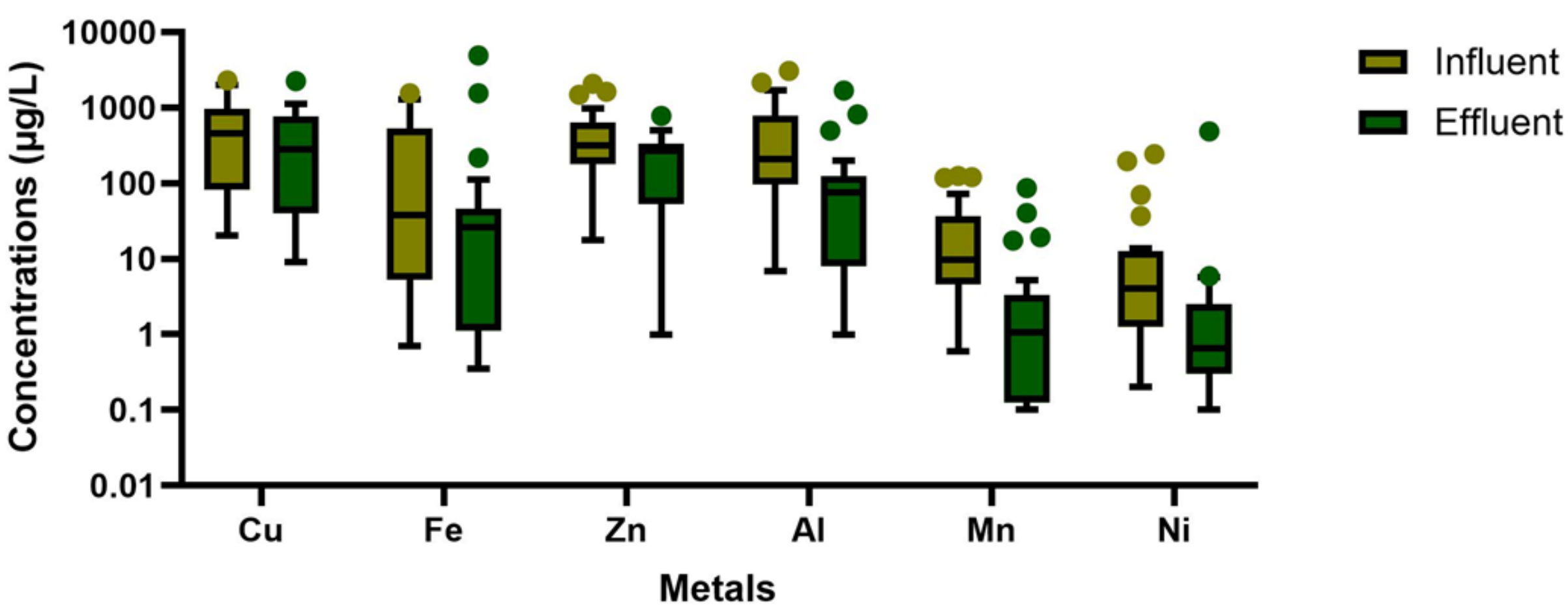

Figure S4. Copper, iron, zinc, aluminum, manganese and nickel in influent and effluent grab samples for 21 hydration stations collected in July 2022. These hydration stations were ones that had their filters extracted with acid.

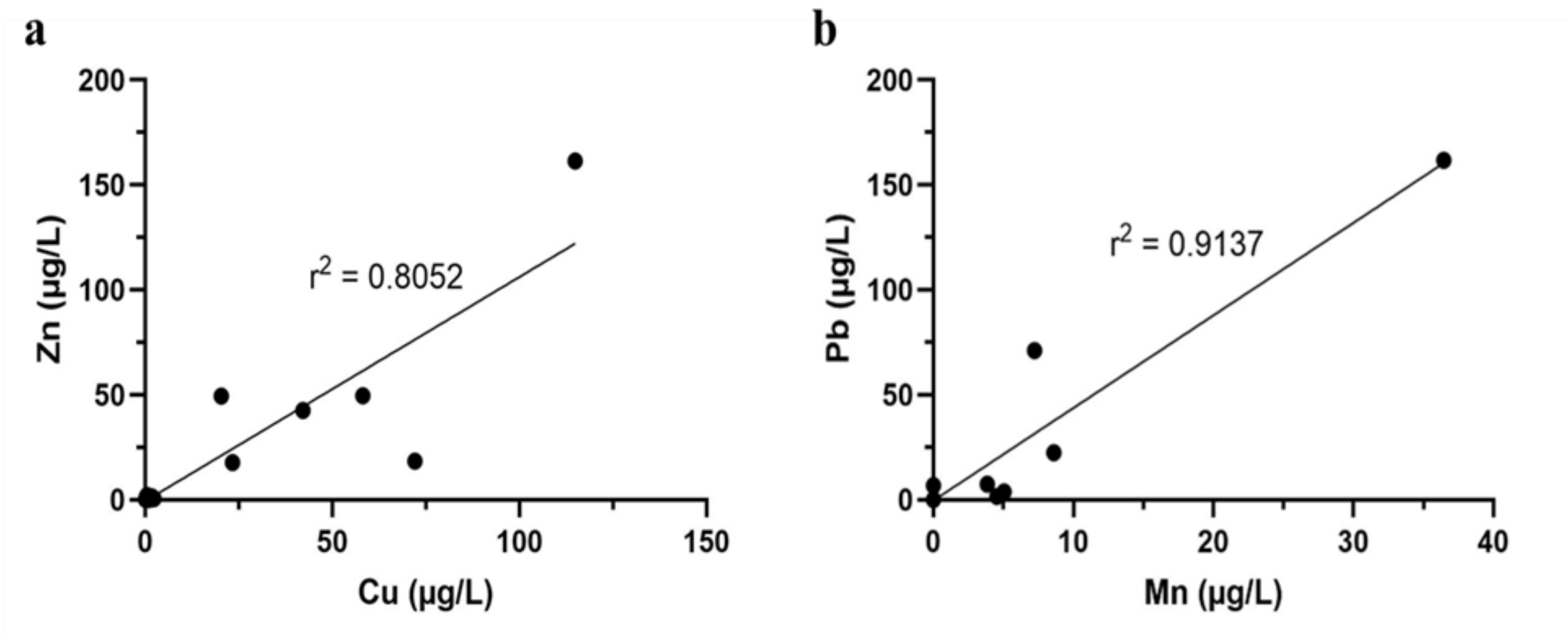

Figure S5. Influent grab sample concentrations of (a) Zn and Cu and (b) Pb and Mn for 21 hydration stations collected in July 2022. These hydration stations were among those whose filters were extracted with acid.

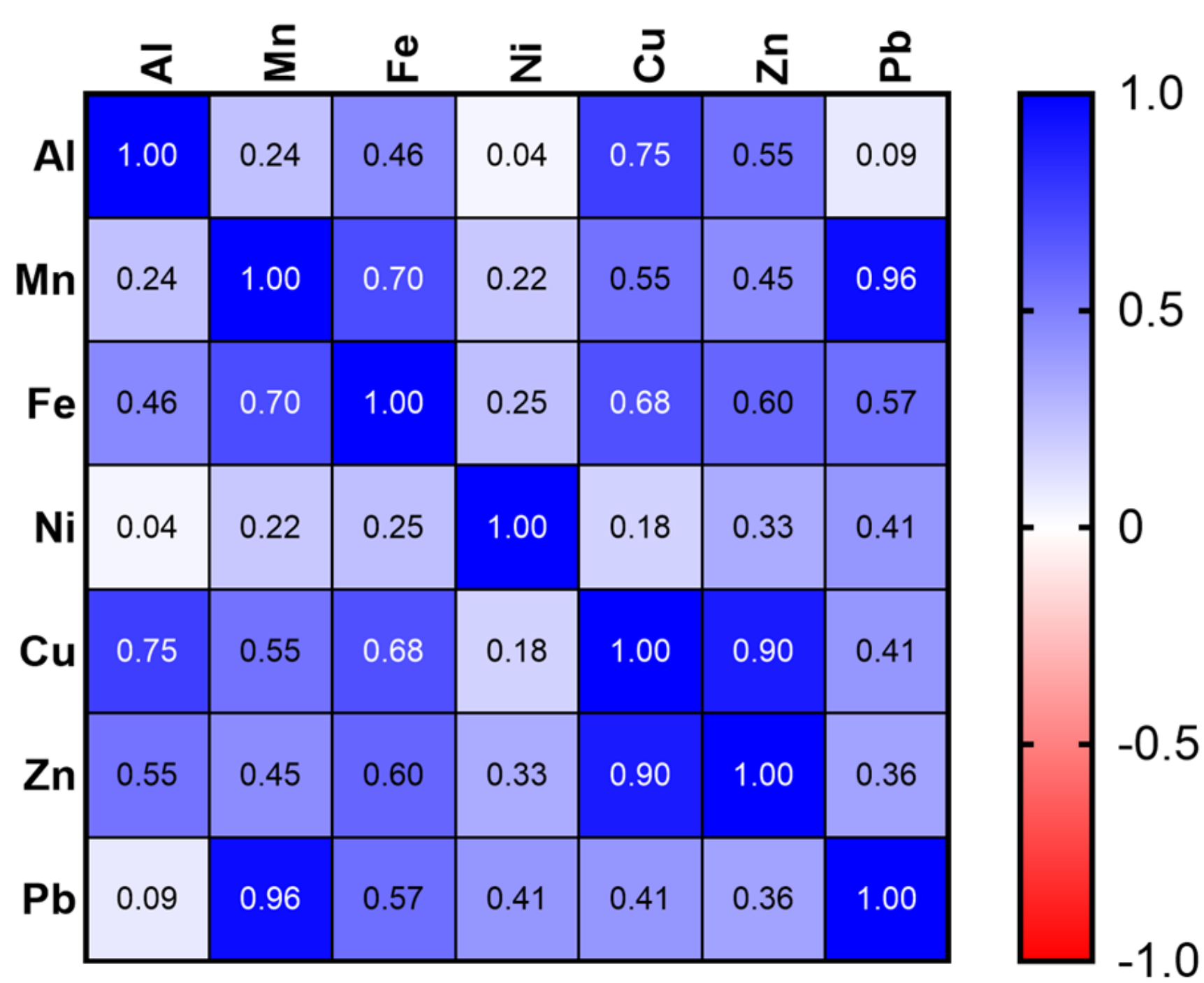

Figure S6. Two-tailed Pearson r correlation coefficients between pairs of metals in influent grab water samples taken in July 2022.

Section S4. Total lead in influent grab samples collected across three field sampling events.

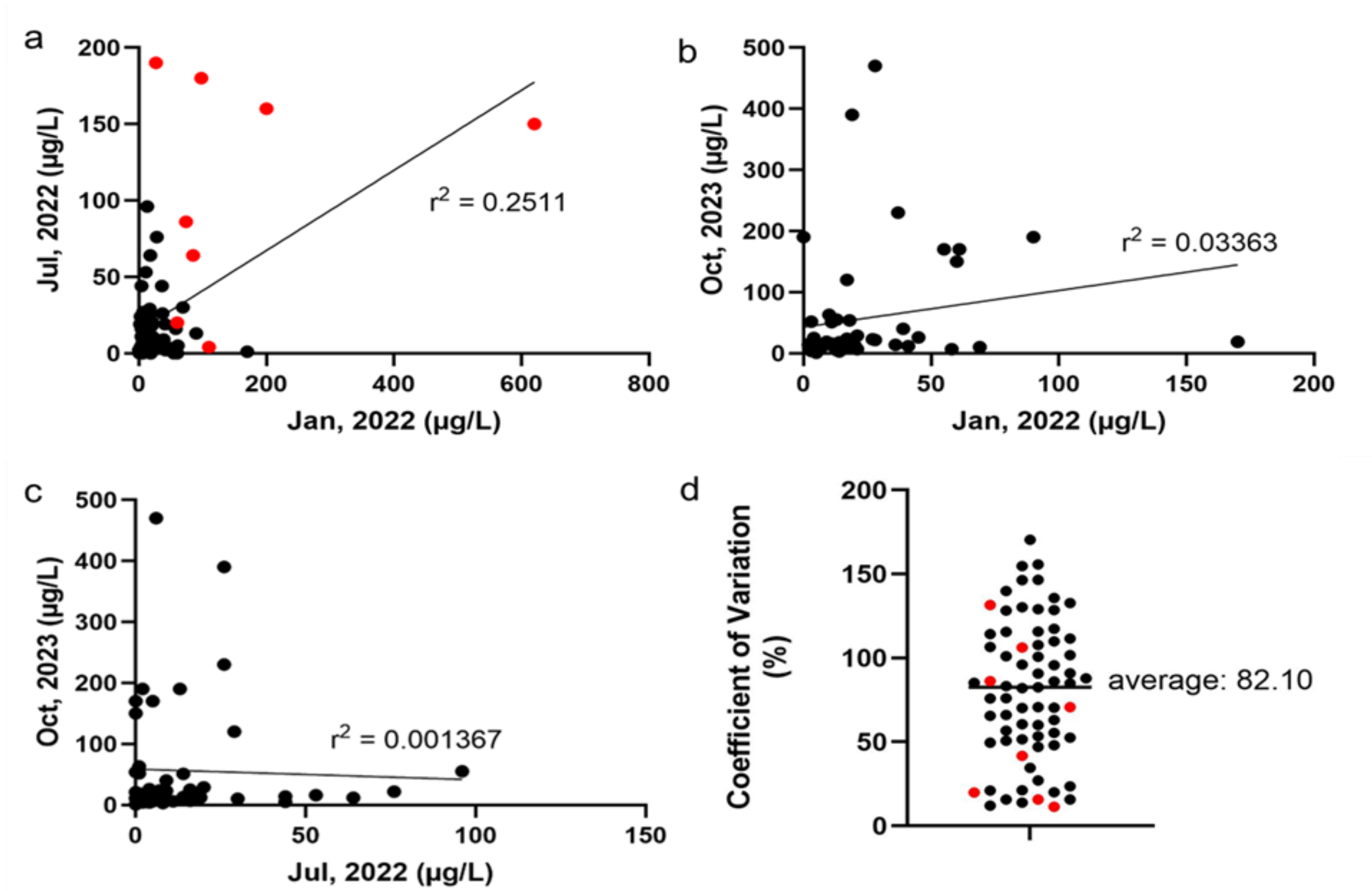

Figure S7. Comparing total lead entering AHS units from two sampling dates: (a) January 2022 and July 2022, (b) January 2022 and October 2023, and (c) July 2022 and October 2023. Coefficient of variation (ratio between standard deviation and mean) was calculated for AHS units that were sampled for at least two times (d). AHS units that were taken sequential samples in October 2023 are colored in red in (a) and (d).

Table S5. Total lead concentrations (μg/L) in influent and effluent water samples during the January 2022, July 2022, and October 2023 field campaigns for all sampled AHS units. Each row represents one hydration station. The first letters are a surrogate indicator for the building where the hydration station was located. The number before the hyphen indicates the floor level at which the hydration station was located. The number after the hyphen refers to an individual hydration station on a certain floor in a certain building. ND= Not Detected (detection limit=1 μg/L). NA= Not Available and occurred when AHS was not operational or accessible. * Indicates samples that field team members may have mislabeled. Bottle-filled water for these locations was resampled on August 8, 2022 by FCS, who reported that all resampled filtered water samples were ND. A-E indicates that the filtered water sample was a five-bottle sequential sample; results are given in order of collection on the following row and are delineated by a comma. † indicates that the total volume of water passed through the filter based on the # of bottles saved and recorded by the AHS exceeded the manufacturer's recommendation of 5,678L. Highlighted rows indicate locations where filters from the July 2022 sampling were analyzed for extractable lead.

	January 2022		July 2022		October 2023	
**ID**	**Influent**	**Effluent**	**Influent**	**Effluent**	**Influent**	**Effluent**
**A1-1**	110	ND	4	ND	A-E	ND
					3, 2, ND, ND, 5	
**A1-2**	60	ND	20	ND	A-E	ND
					62, 27, 11, 2, 1	
**A1-3**	620	ND	150	ND	A-E	ND
					490, 130, 25, 14, 21	
**A1-4**	85	ND	64	ND†	A-E	ND
					170, 6, 2, 3, 18	
**B1-1**	18	ND	ND	ND	54	ND
**B1-2**	9	ND	8	ND	19	ND
**B1-3**	3	ND	1	ND	52	ND
**B1-4**	74	ND	ND*	87*	A-E	NA
					20, 4, ND, ND, ND	
**C0-1**	11	ND	53	ND	16	ND
**C1-1**	20	ND	18	ND	13	ND
**C1-2**	4	ND	16	ND	25	ND

Table S5 (continued)

ID	January 2022		July 2022		October 2023	
	Influent	Effluent	Influent	Effluent	Influent	Effluent
**C1-3**	4	ND	5	ND	6	ND
**C1-4**	4	ND	11	ND	6	ND
**C1-5**	36	ND	44	ND	14	ND
**C1-6**	4	ND	44	ND	5	ND
**C1-7**	28	ND	76	ND	22	ND
**C1-8**	4	ND	ND	ND	21	ND
**C1-9**	21	ND	11	ND	7	ND
**C1-10**	14	ND	18	ND	18	ND
**C1-11**	2	ND	19	ND†	13	NA
**C1-12**	19	ND	ND	ND	NA	NA
**C1-13**	3	ND	24	ND	NA	NA
**C1-14**	69	ND	30	ND	10	ND
**C1-15**	27	ND	190	ND	A-E<br>8, ND, ND, ND, ND	ND
**D1-1**	55	ND	ND*	27*	170	ND
**D1-2**	45	NA	4	ND	26	ND
**D1-3**	7	ND	3	ND	13	ND
**E0-1**	6	ND	1	ND	NA	NA
**E1-1**	16	ND	8	ND	NA	NA
**E2-1**	5	ND	2	ND	NA	NA
**F1-1**	3	ND	2	ND	6	ND
**F1-2**	10	ND	1	ND	63	ND

Table S5 (continued)

ID	January 2022		July 2022		October 2023	
	Influent	Effluent	Influent	Effluent	Influent	Effluent
**F1-3**	19	ND	26	ND	390	ND
**F1-4**	90	ND	13	ND	190	ND
**F1-5**	6	ND	ND	ND	11	ND
**F1-6**	34	ND	4	ND	11	ND
**F2-1**	5	ND	2	ND	16	ND
**F2-2**	170	ND	1	ND	19	ND
**F2-3**	58	ND	16	ND	7	ND
**F3-1**	ND	ND	2	ND	190	ND
**G0-1**	17	ND	7	ND	24	ND
**G0-2**	61	ND	5	ND	170	ND
**G1-1**	98	ND	180	ND	A-E	ND
					6, 10, ND, 3, ND	
**G1-2**	6	ND	8	ND	8	ND
**G1-3**	37	ND	26	ND	230	NA
**G1-4**	NA	NA	14	ND	10	ND
**G1-5**	27	ND	9	ND	23	ND
**G1-6**	11	ND	14	ND	51	3
**G2-1**	200	ND	160	ND	A-E	ND
**G2-2**	13	ND	96	ND	55	ND
**G3-1**	17	NA	29	ND	120	NA
**G3-2**	18	ND	64	ND	12	ND
					1, 2, 35, 2, 4	
**G2-2**	13	ND	96	ND	55	ND
**G3-1**	17	NA	29	ND	120	NA

Table S5 (continued)

	January 2022		July 2022		October 2023	
**ID**	**Influent**	**Effluent**	**Influent**	**Effluent**	**Influent**	**Effluent**
**G3-2**	18	ND	64	ND	12	ND
**H1-1**	3	ND	4	ND	4	ND
**I1-1**	11	ND	14	ND	13	ND
**I1-2**	41	ND	19	ND	12	ND
**I1-3**	7	ND	6	ND	10	ND
**J1-1**	11	NA	24	1	NA	NA
**J1-2**	42	ND	2	ND	NA	NA
**J1-3**	1	ND	ND	ND	NA	NA
**J1-4**	3	ND	1	ND	NA	NA
**J1-5**	7	ND	27	ND	NA	NA
**J1-6**	8	ND	2	ND	NA	NA
**K1**	14	ND	NA	NA	NA	NA
**K2**	1	ND	NA	NA	NA	NA
**K3**	2	ND	NA	NA	NA	NA
**K4**	2	ND	NA	NA	NA	NA
**L1-1**	4	ND	2	ND	4	ND
**L1-2**	21	ND	20	2	29	1
**L1-3**	2	ND	1	ND	15	ND
**L1-4**	14	ND	8	ND	3	ND

Table S5 (continued)

ID	January 2022		July 2022		October 2023	
	Influent	Effluent	Influent	Effluent	Influent	Effluent
**M1-1**	5	ND	ND	ND†	1	ND
**M1-2**	60	ND	ND	ND	150	NA
**M1-3**	28	ND	6	ND	470	ND
**M1-4**	39	ND	9	ND	40	ND
**M1-5**	13	ND	2	ND	5	ND

Section S5. Individual total lead concentrations in sequential samples for all eight AHS units.

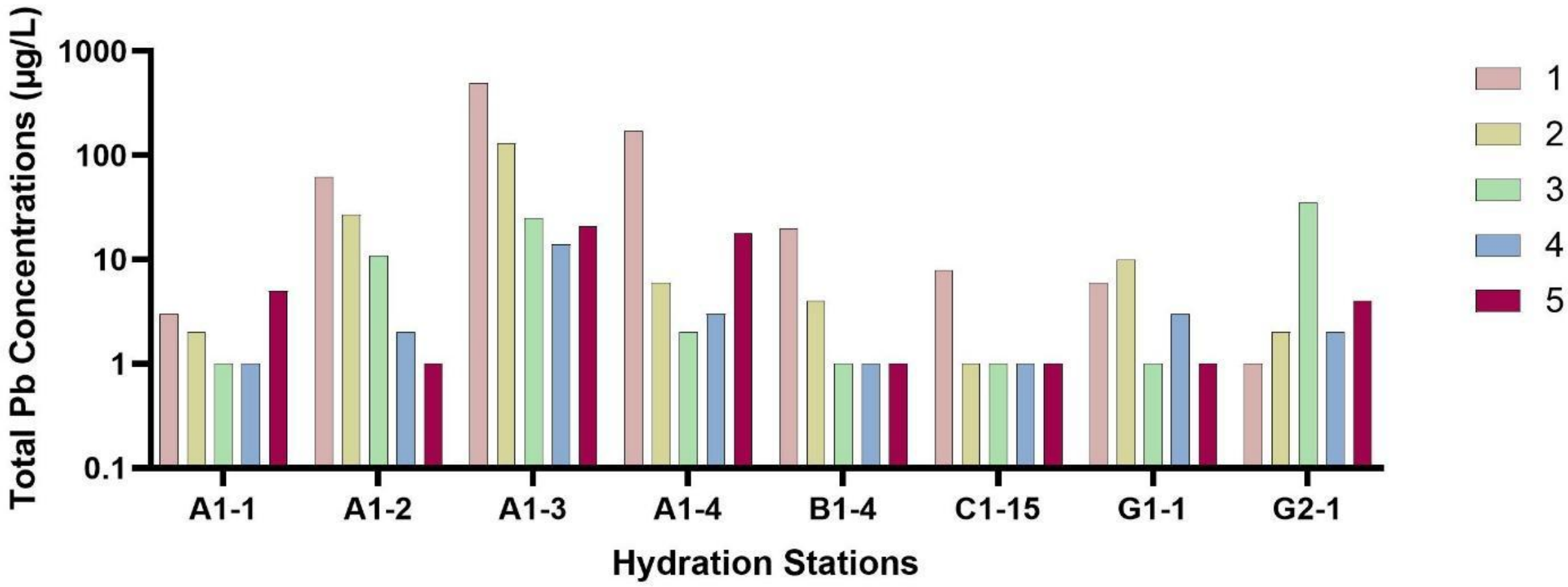

Figure S8. Individual influent total lead concentrations in sequential samples taken in October 2023 for eight AHS units that had high lead levels from January 2022 and July 2022 sampling events (see Table S5). For samples that had undetected lead levels, 1 µg/L was entered as the total lead concentration.

Section S6: Lead extraction profiles of POU filters removed from hydration stations.

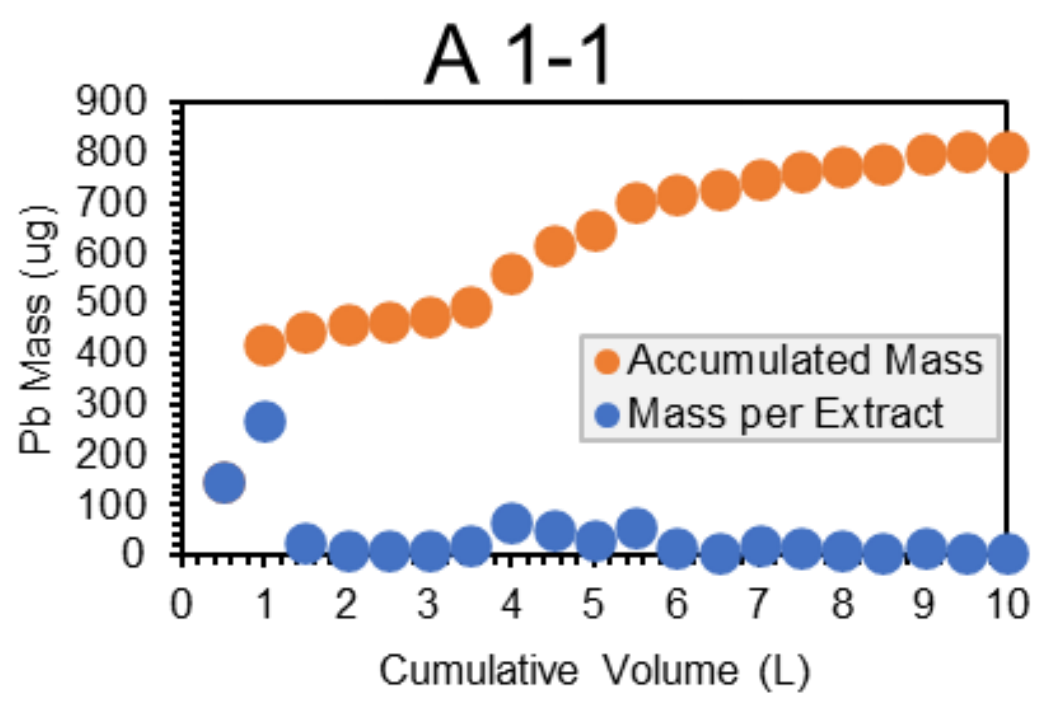

(a)

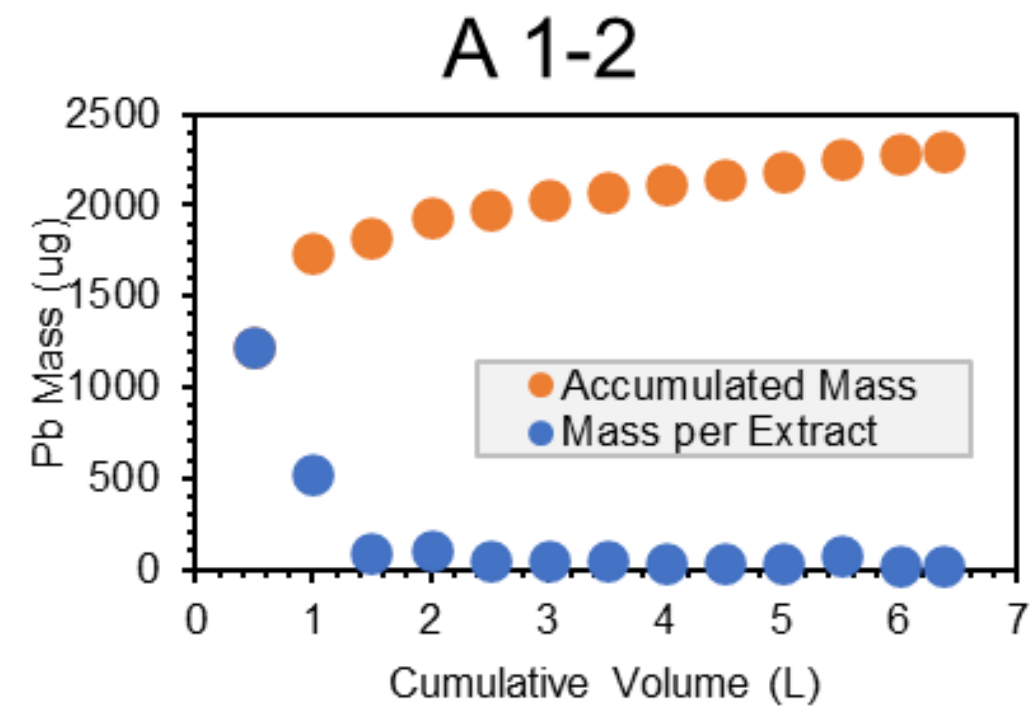

(b)

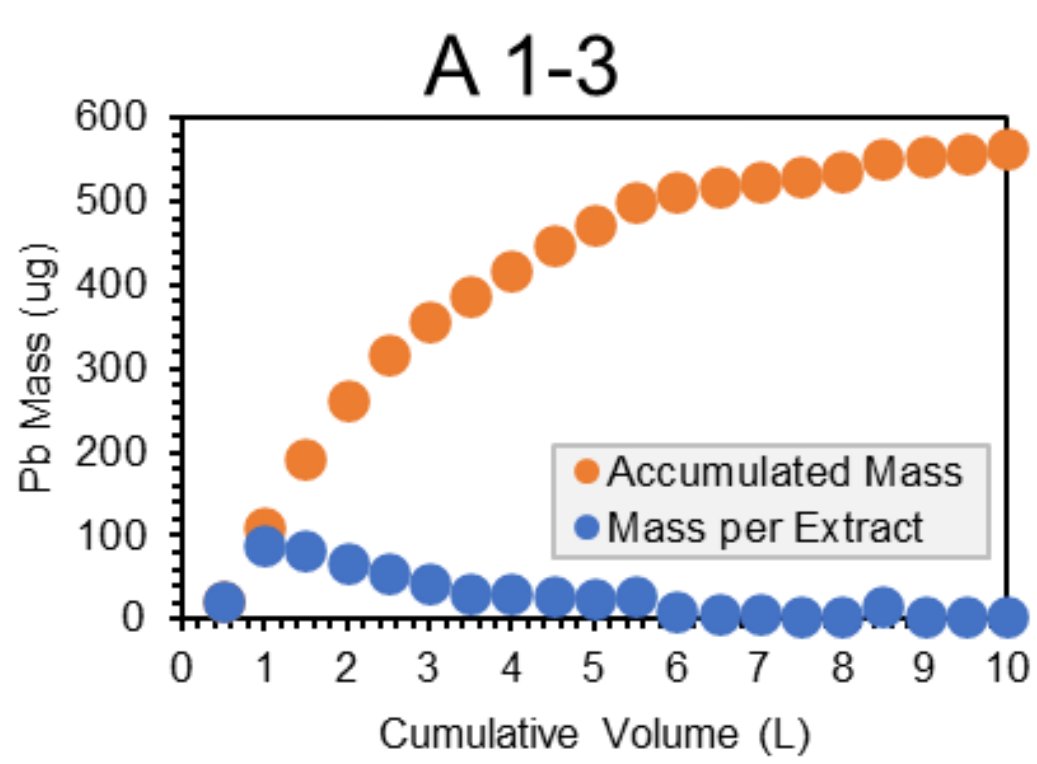

(c)

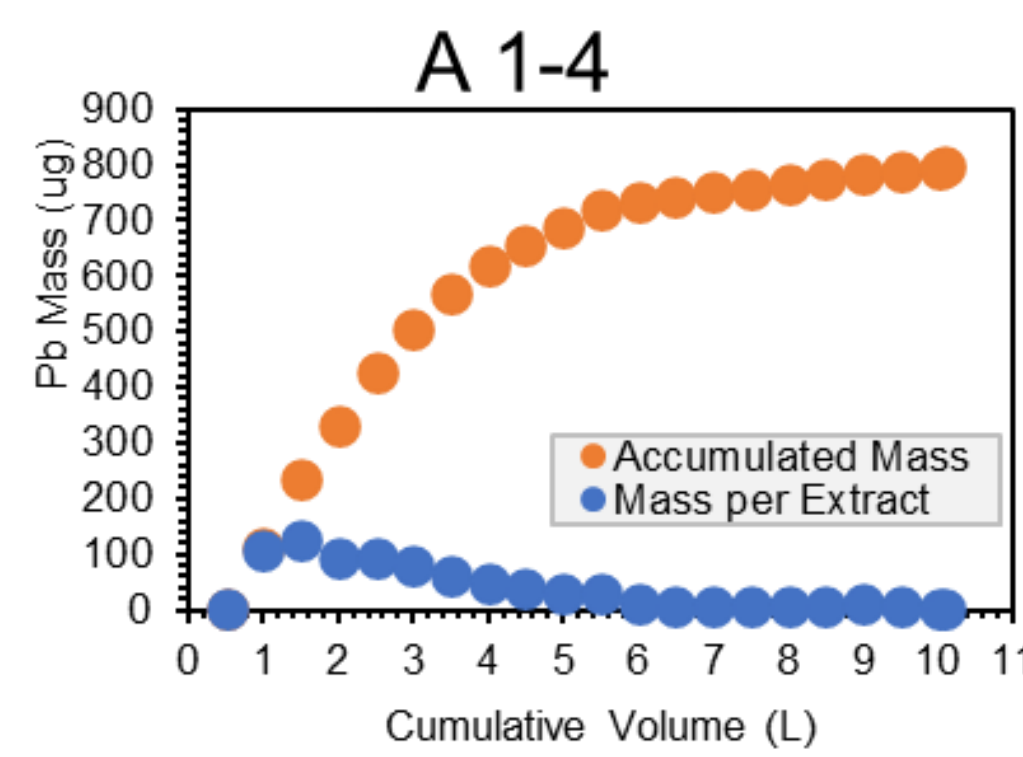

(d)

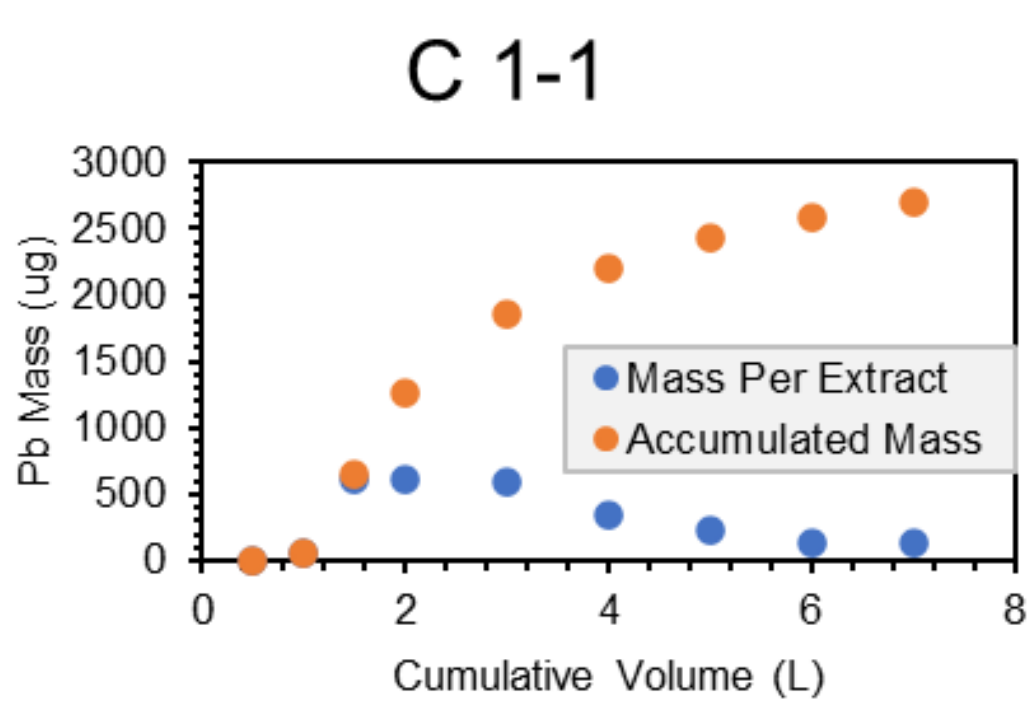

(e)

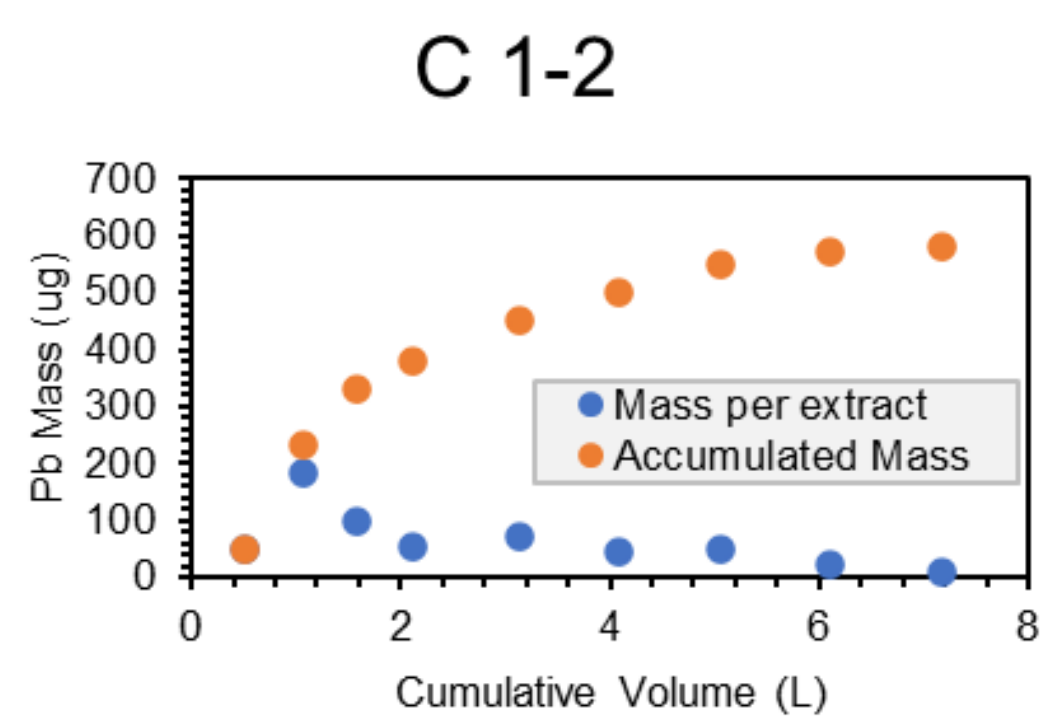

(f)

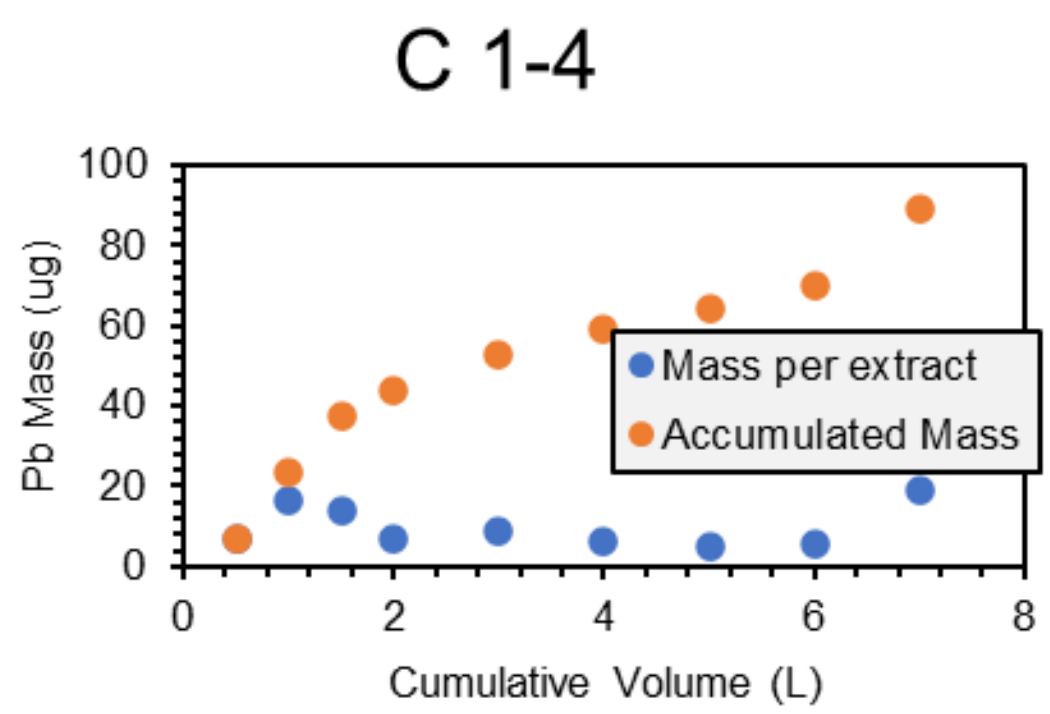
C 1-4
Pb Mass (ug)
Mass per extract
Accumulated Mass
Cumulative Volume (L)

(g)

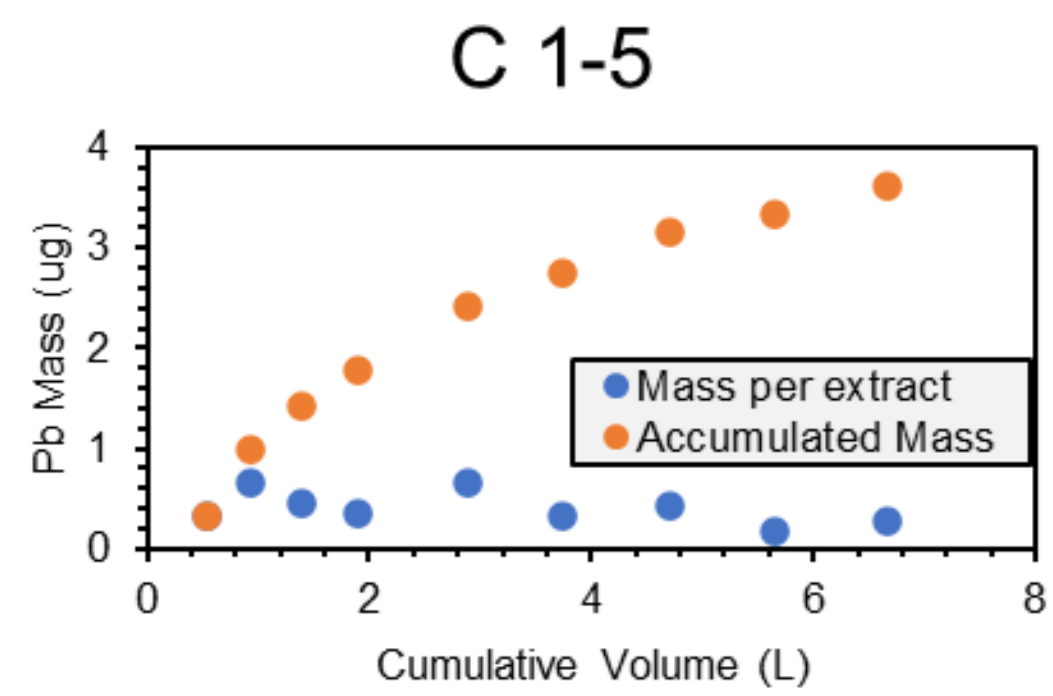
C 1-5
Pb Mass (ug)
Mass per extract
Accumulated Mass
Cumulative Volume (L)

(h)

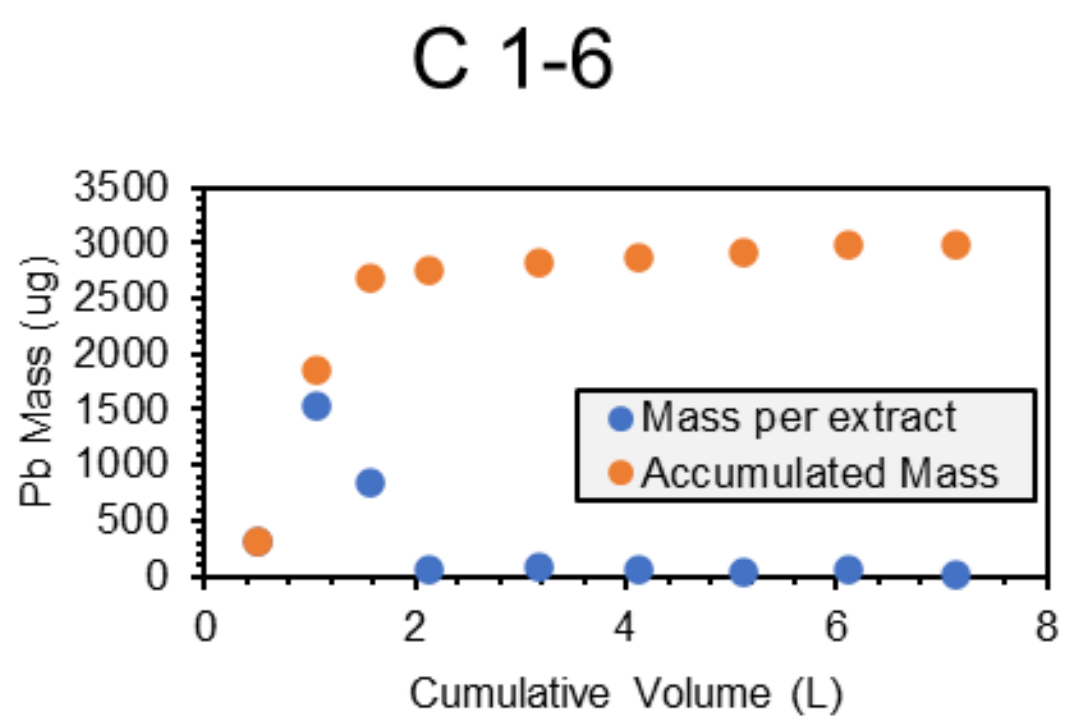
C 1-6
Pb Mass (ug)
Mass per extract
Accumulated Mass
Cumulative Volume (L)

(i)

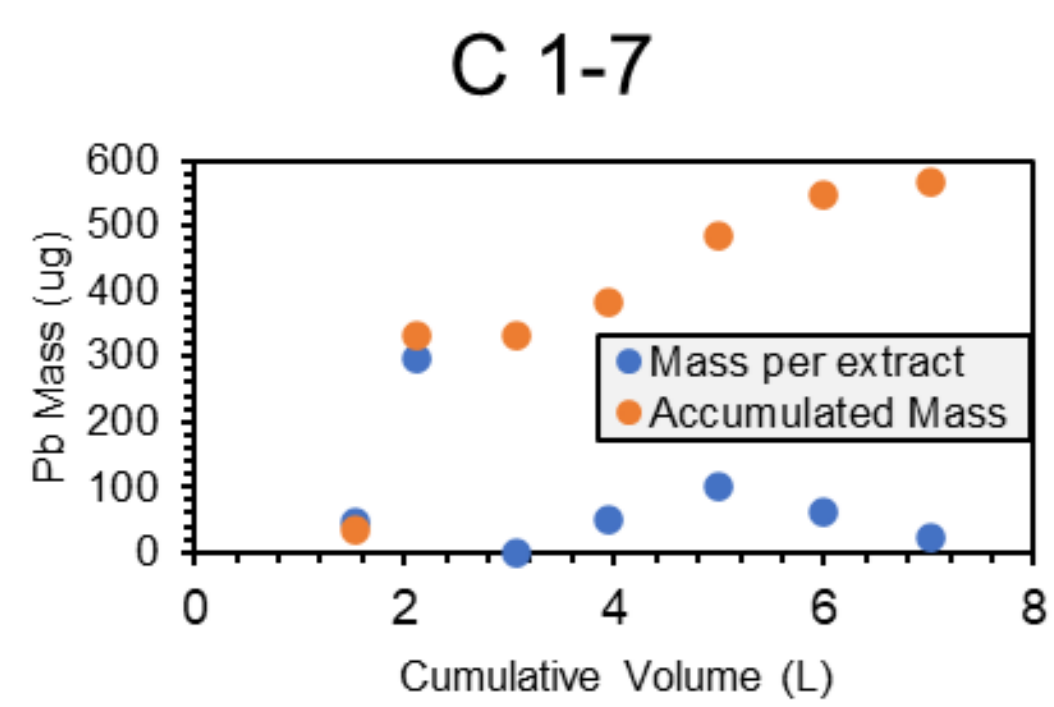
C 1-7
Pb Mass (ug)
Mass per extract
Accumulated Mass
Cumulative Volume (L)

(j)

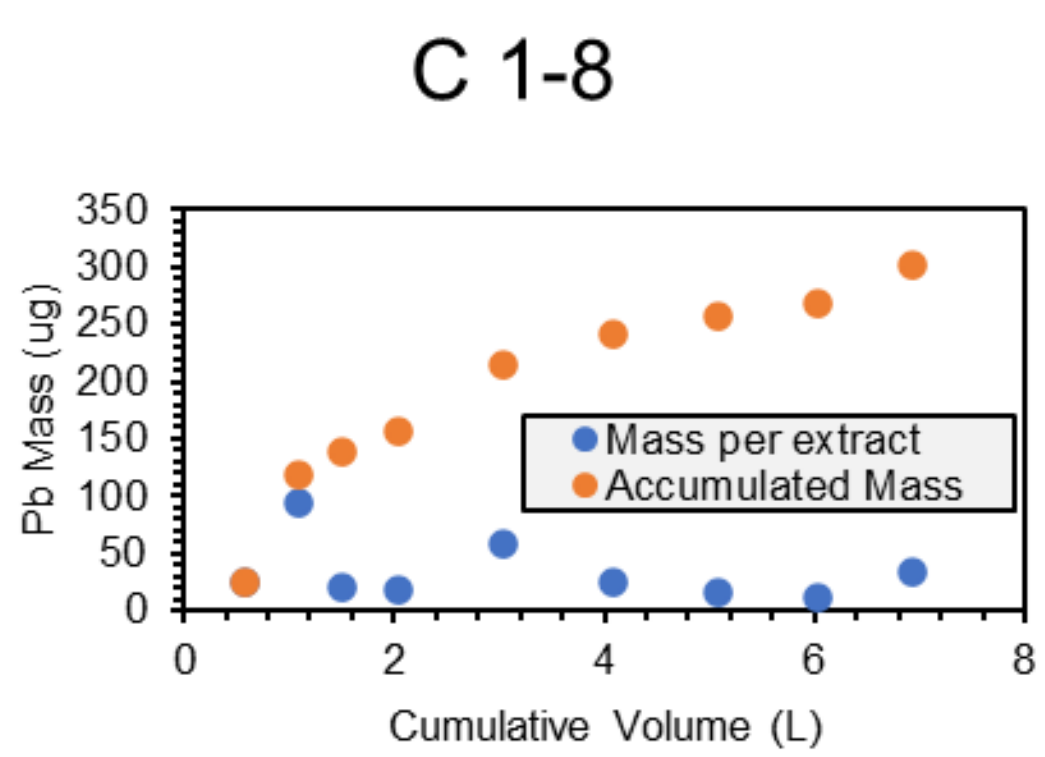
C 1-8
Pb Mass (ug)
Mass per extract
Accumulated Mass
Cumulative Volume (L)

(k)

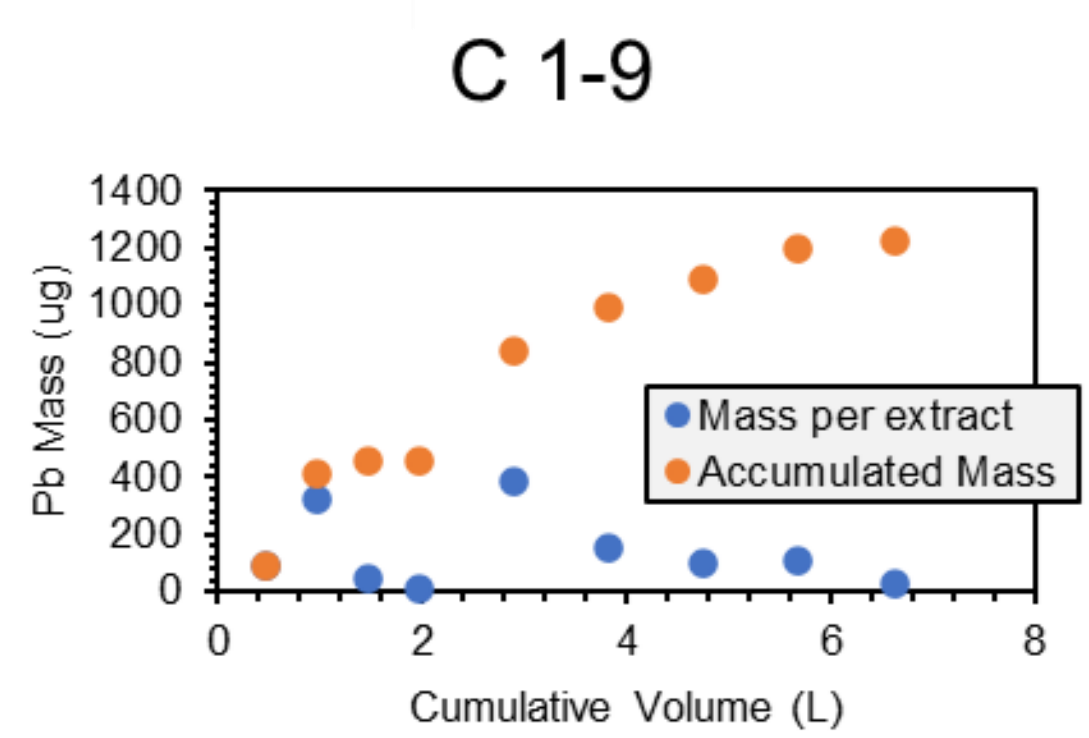
C 1-9
Pb Mass (ug)
Mass per extract
Accumulated Mass
Cumulative Volume (L)

(l)

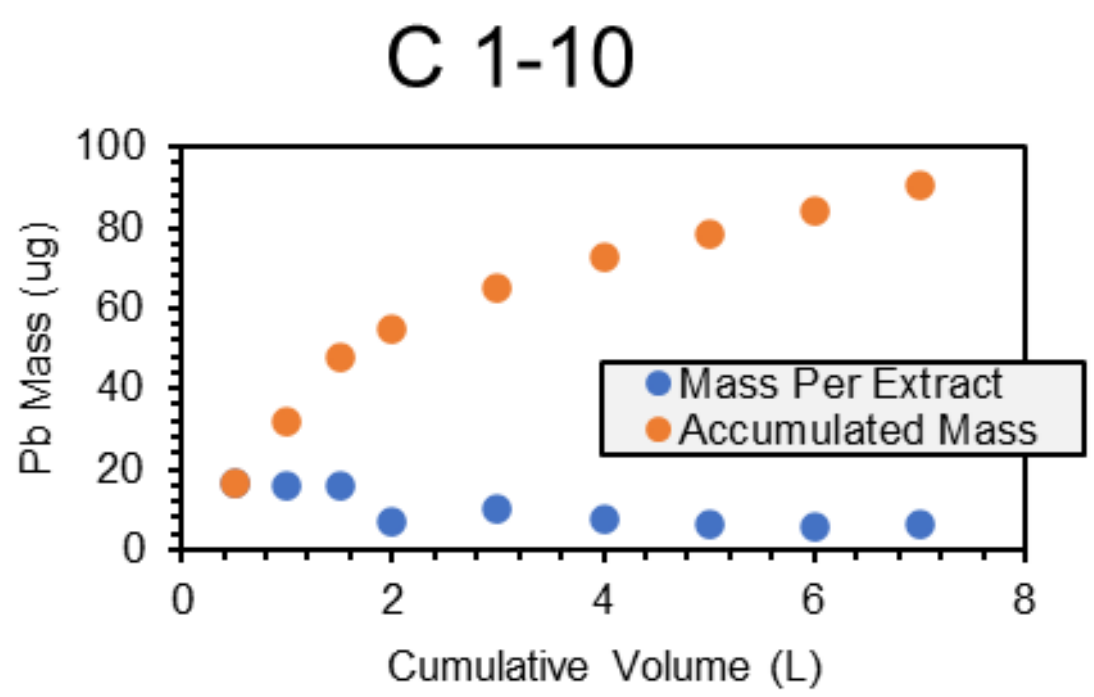
C 1-10
Pb Mass (ug)
Cumulative Volume (L)
Mass Per Extract
Accumulated Mass

(m)

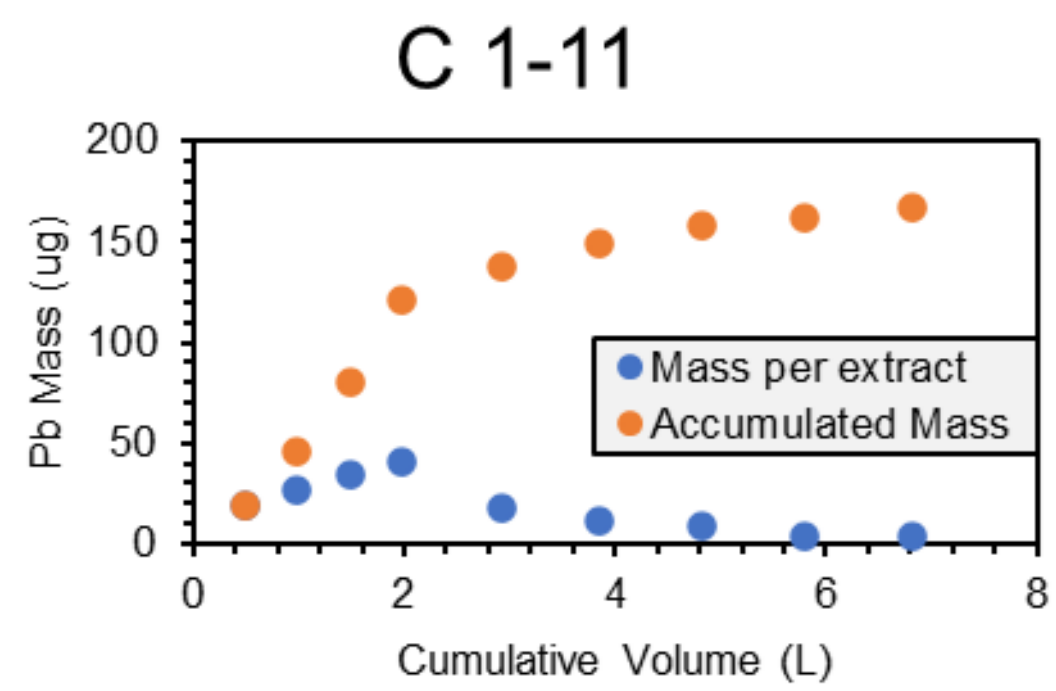
C 1-11
Pb Mass (ug)
Cumulative Volume (L)
Mass per extract
Accumulated Mass

(n)

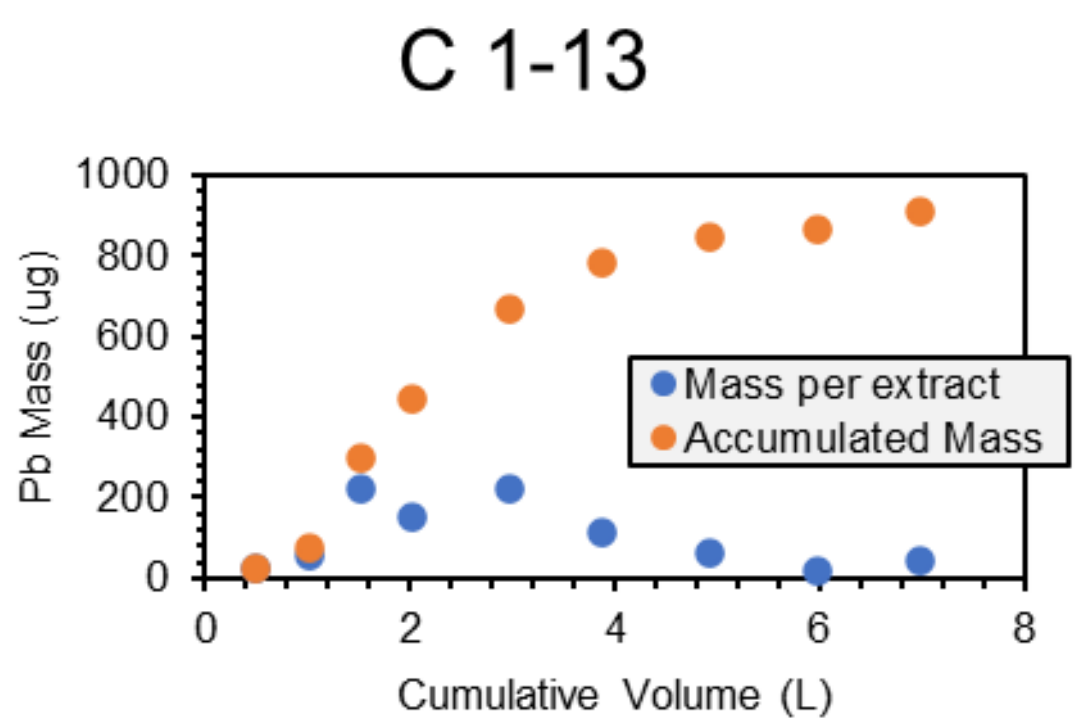
C 1-13
Pb Mass (ug)
Cumulative Volume (L)
Mass per extract
Accumulated Mass

(o)

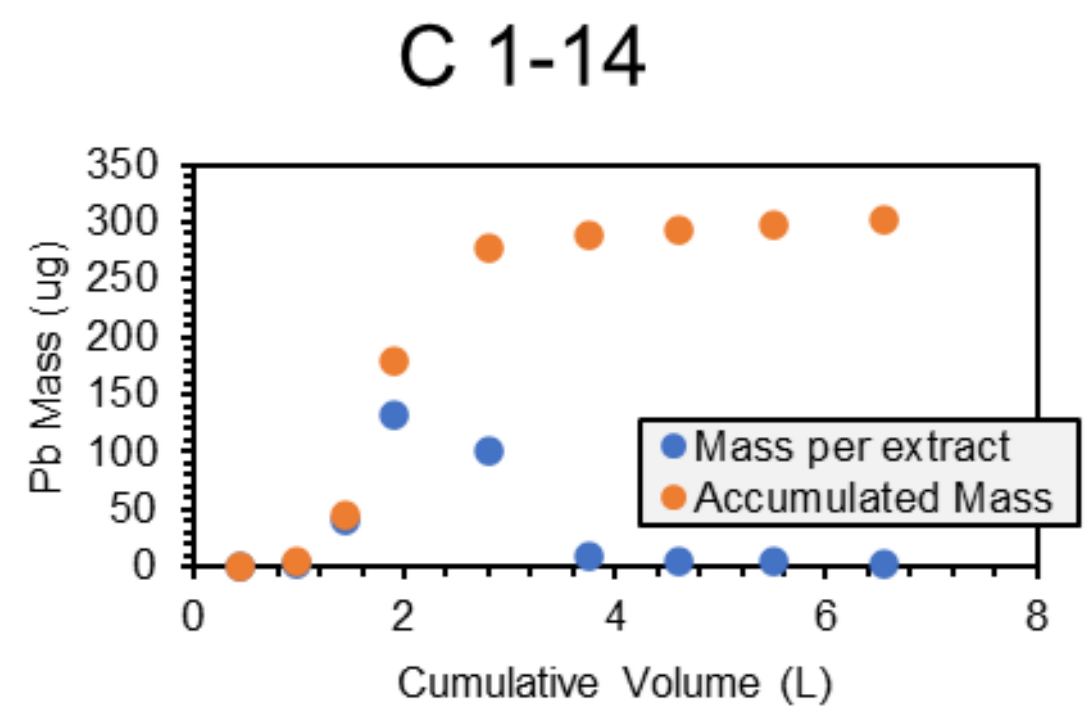
C 1-14
Pb Mass (ug)
Cumulative Volume (L)
Mass per extract
Accumulated Mass

(p)

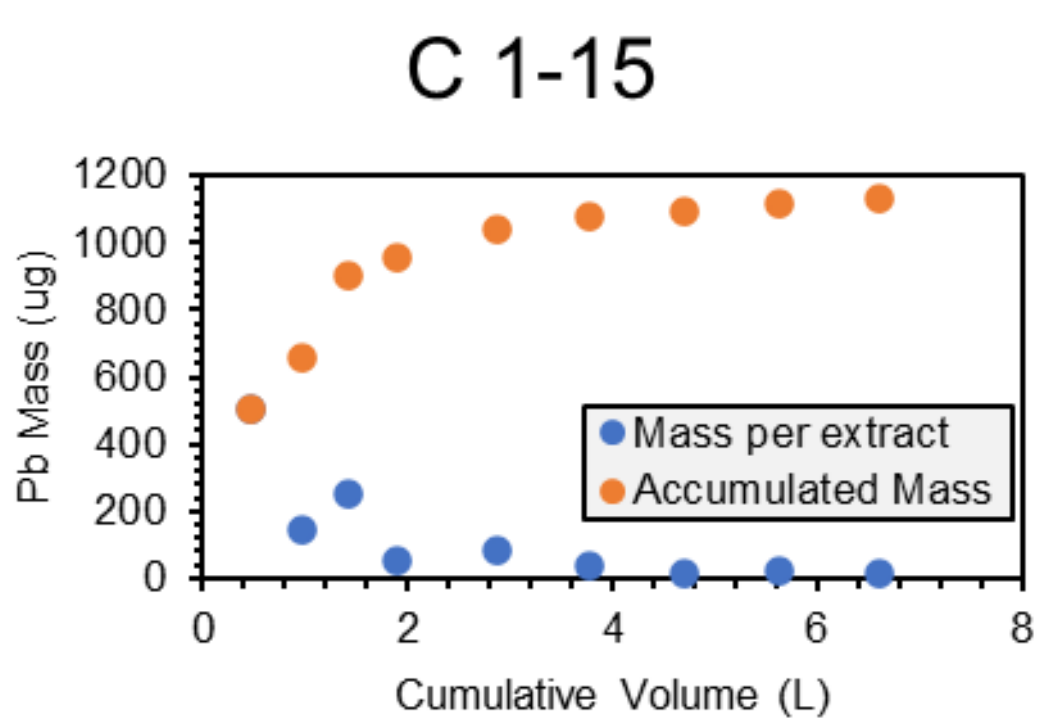
C 1-15
Pb Mass (ug)
Cumulative Volume (L)
Mass per extract
Accumulated Mass

(q)

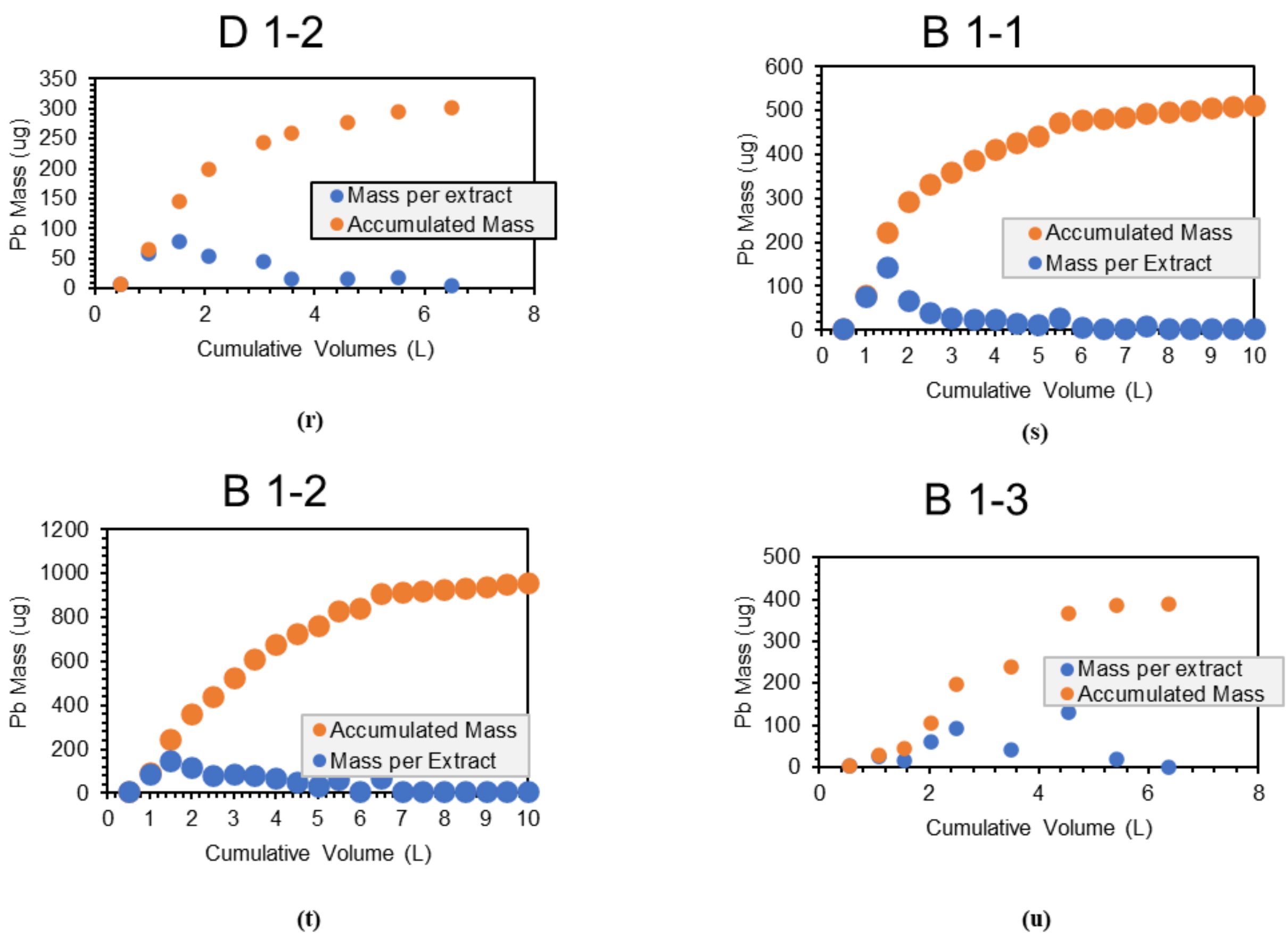

Figure S9. Lead extraction profiles of POU filters harvested during July 2022 field campaigns. Each graph represents the lead extraction profile of an individual filter as a function of cumulative volume of nitric acid as an extractant. The title of each graph indicates the filter location.

Section S7. Single grab and volume-averaged concentrations of metals other than lead.

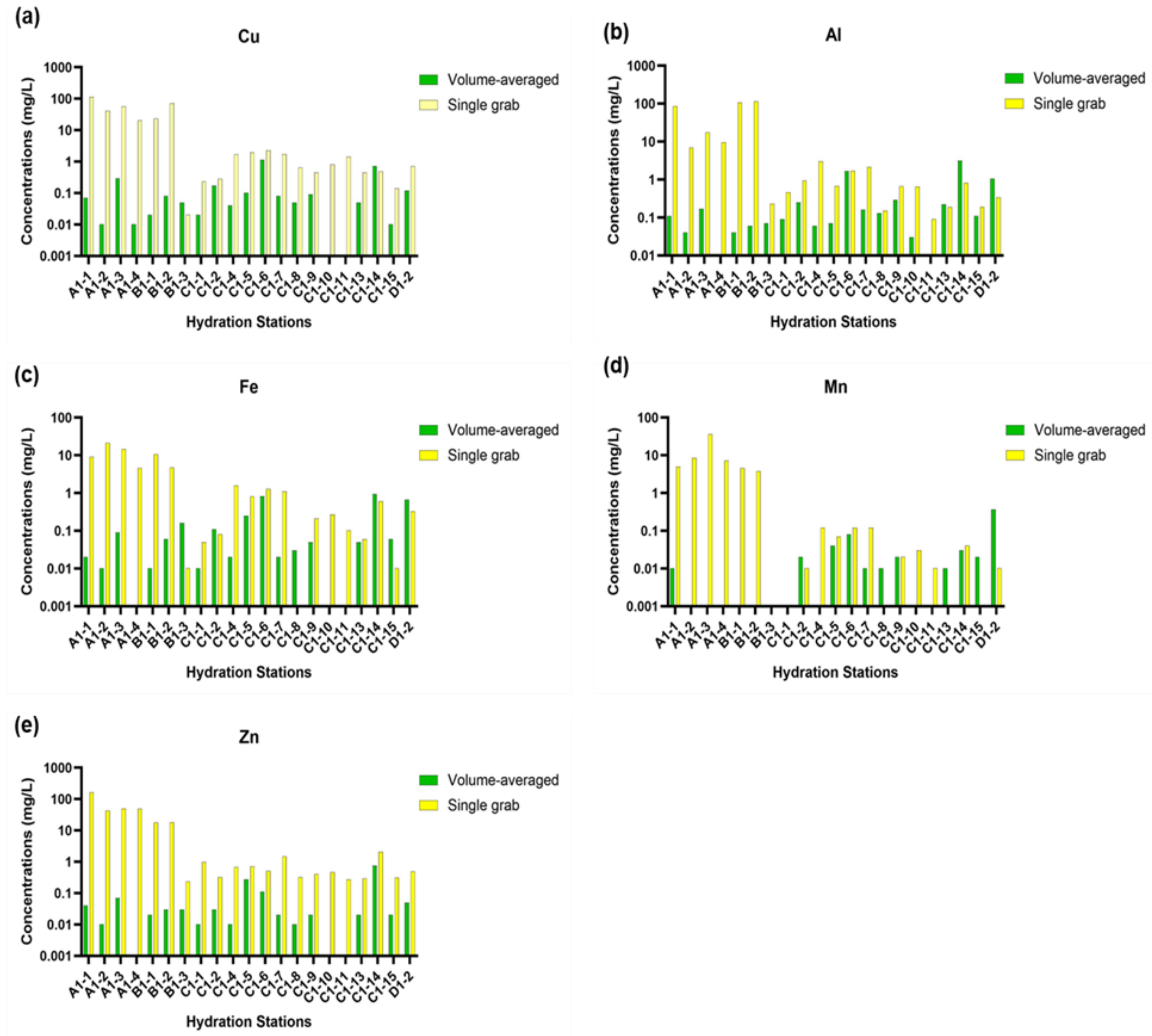

Figure S10. Volume-averaged and influent grab sample Cu (a), Al (b), Fe (c), Mn (d), Zn (e) concentrations in upstream water entering hydration stations

Section S8. Correlation between masses of total extracted metal.

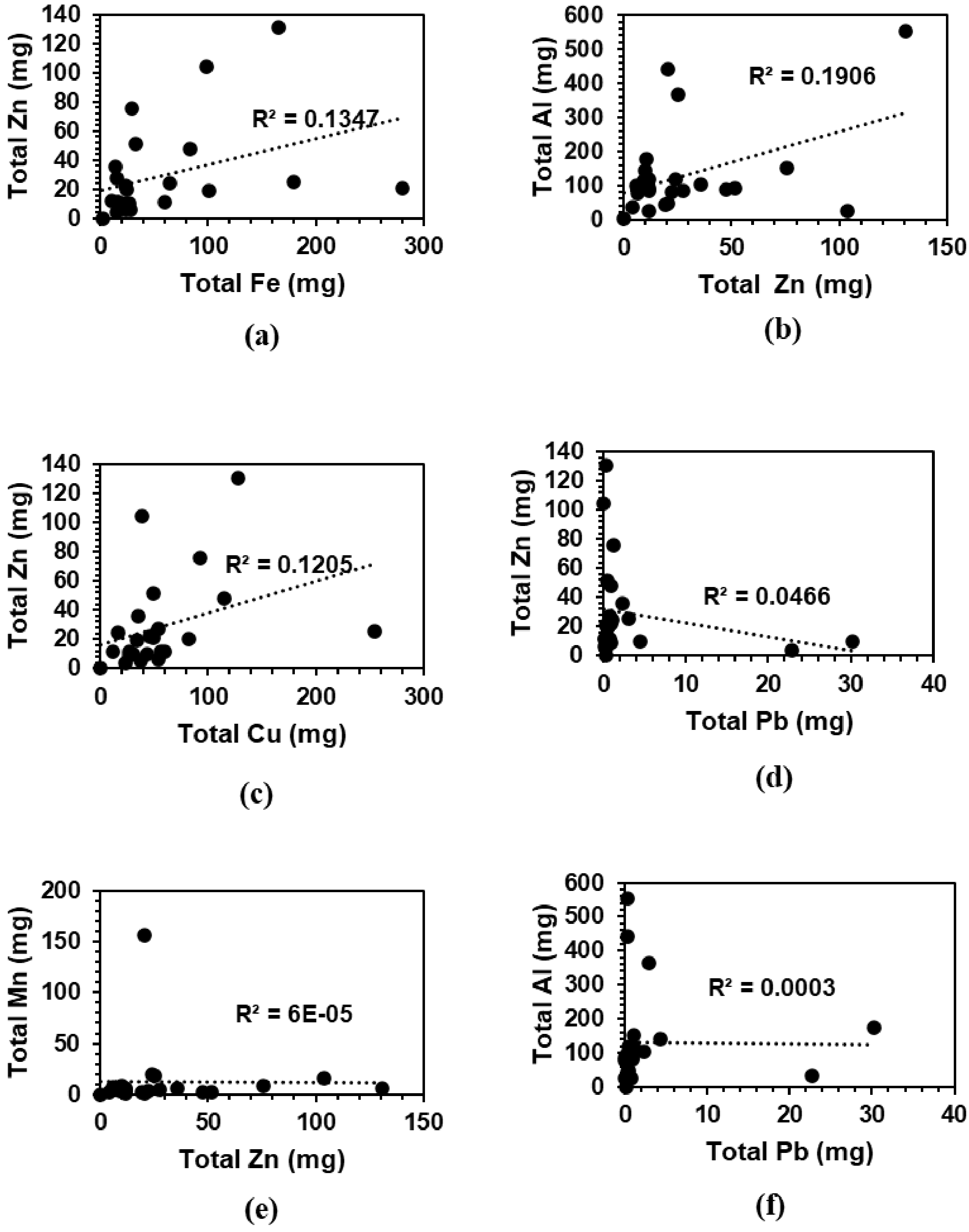

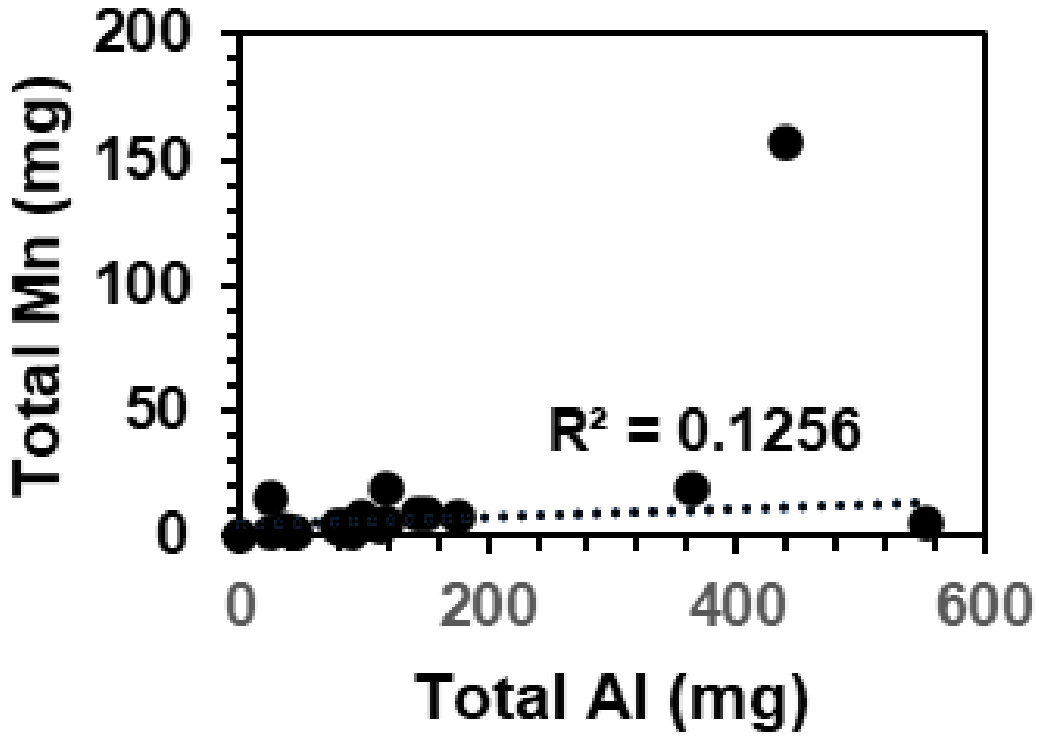
Total Mn (mg)
Total Al (mg)
R^2 = 0.1256
0
50
100
150
200
0
200
400
600

(g)

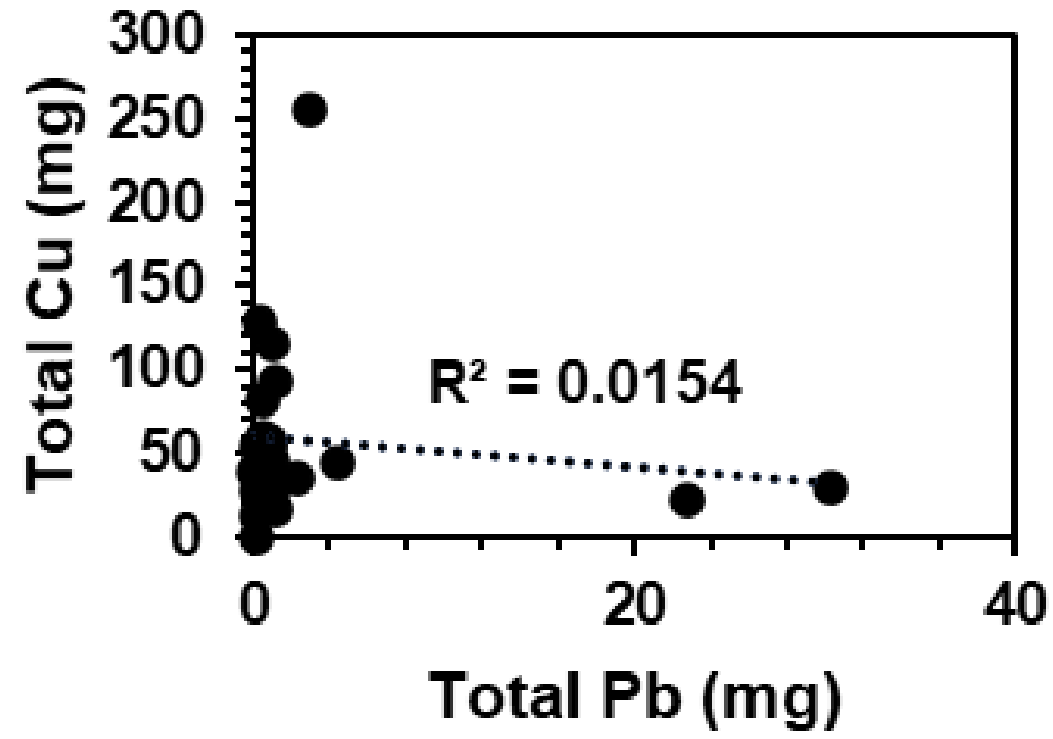
Total Cu (mg)
Total Pb (mg)
R^2 = 0.0154
0
50
100
150
200
250
300
0
20
40

(h)

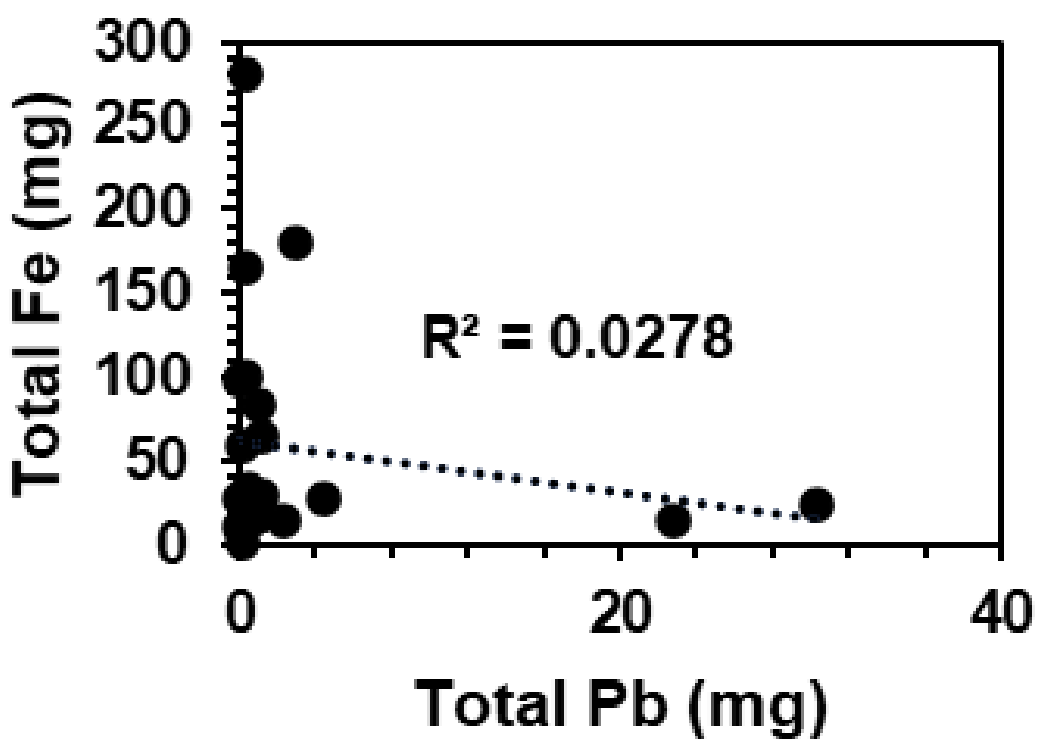
Total Fe (mg)
Total Pb (mg)
R^2 = 0.0278
0
50
100
150
200
250
300
0
20
40

(i)

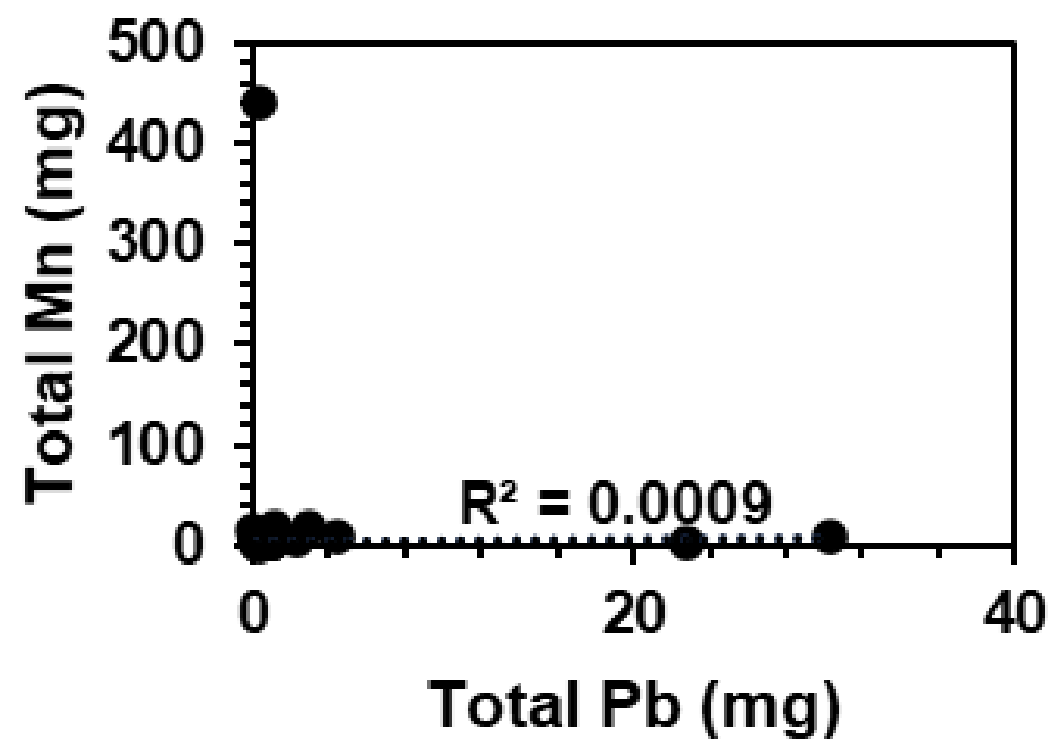
Total Mn (mg)
Total Pb (mg)
R^2 = 0.0009
0
100
200
300
400
500
0
20
40

(j)

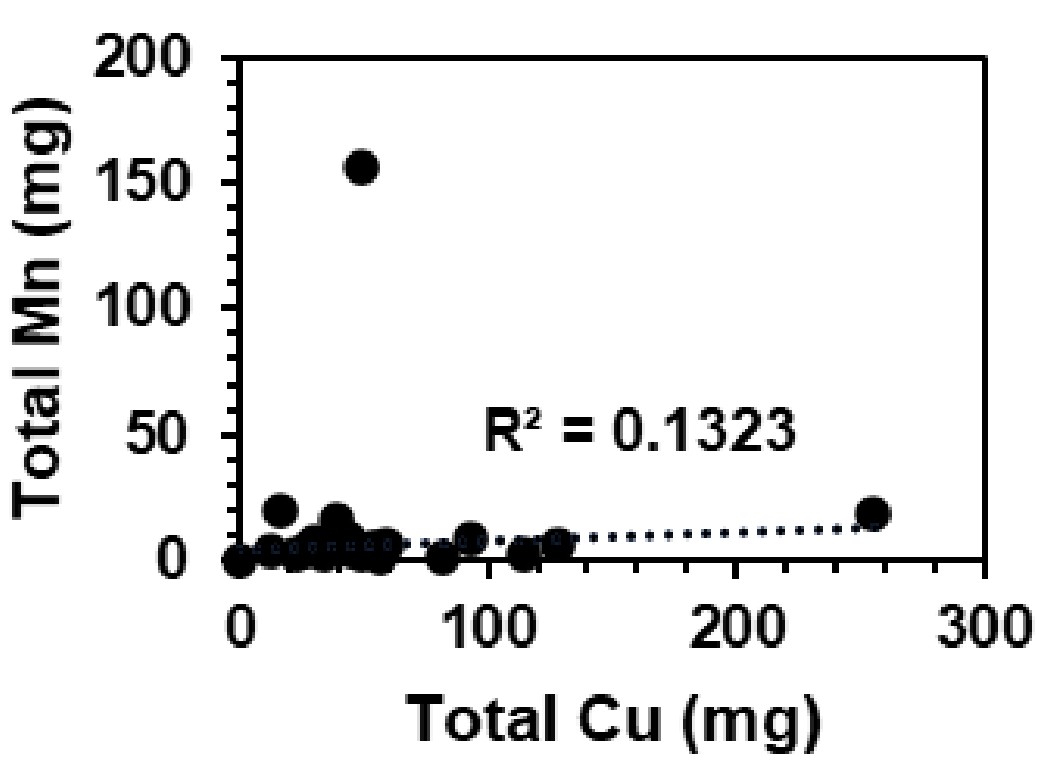
Total Mn (mg)
Total Cu (mg)
R^2 = 0.1323
0
50
100
150
200
0
100
200
300

(k)

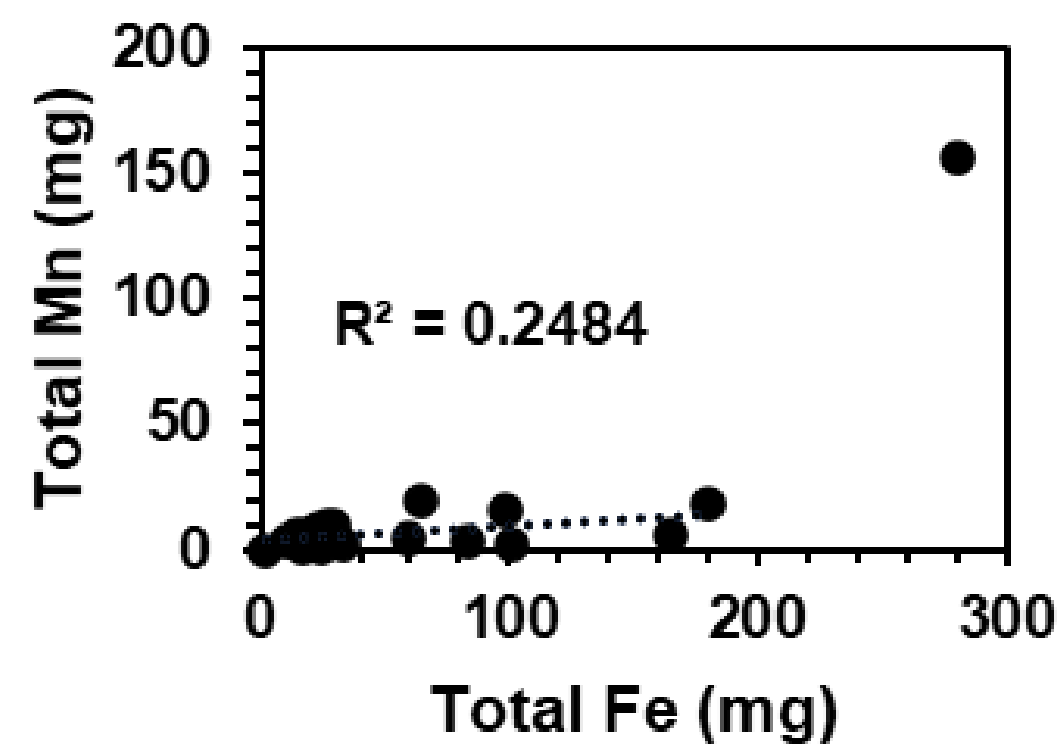
Total Mn (mg)
Total Fe (mg)
R^2 = 0.2484
0
50
100
150
200
0
100
200
300

(l)

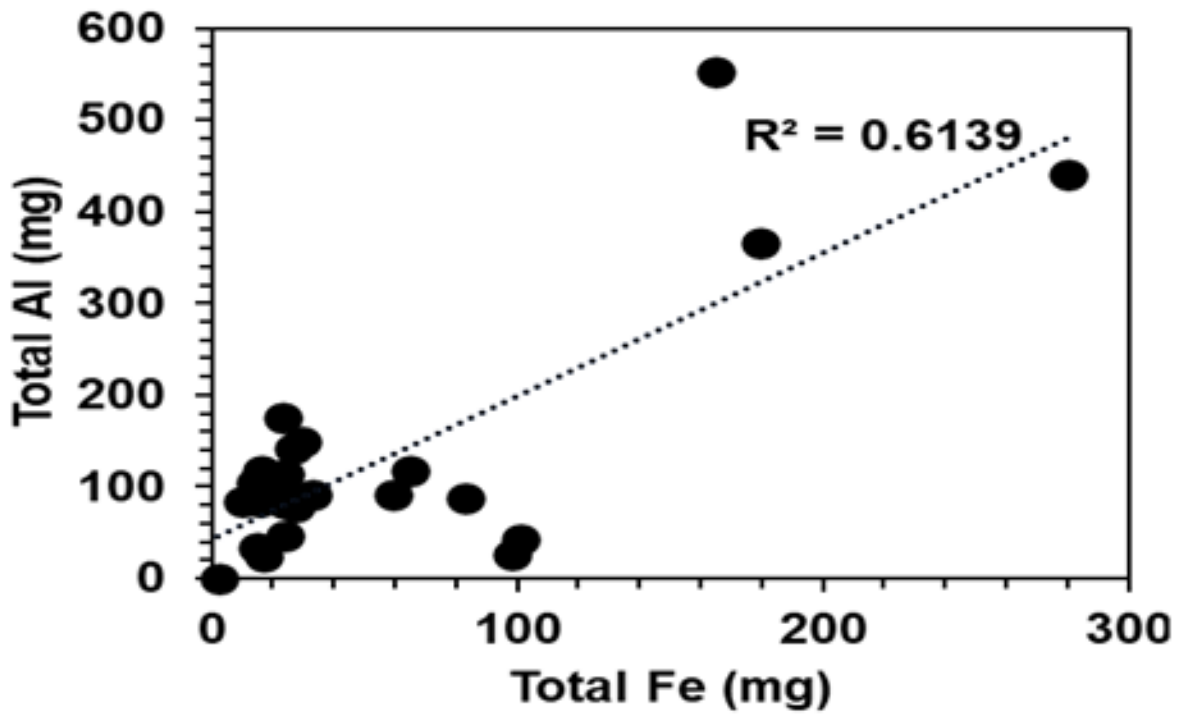

(m)

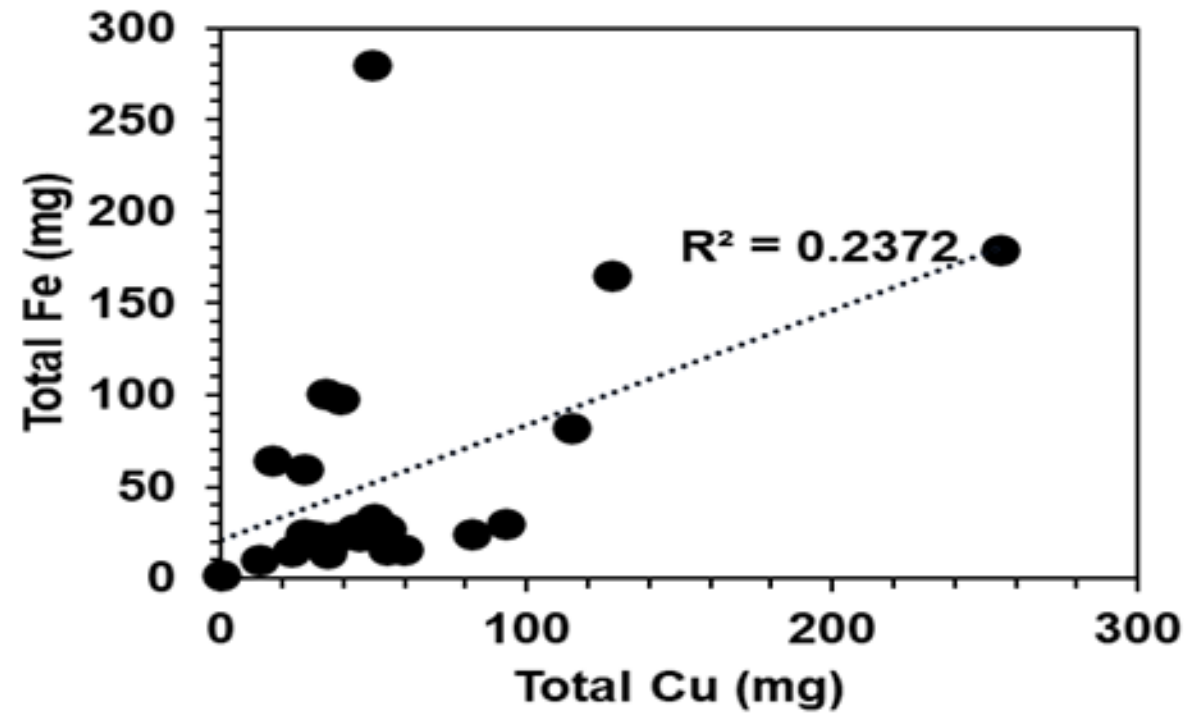

(n)

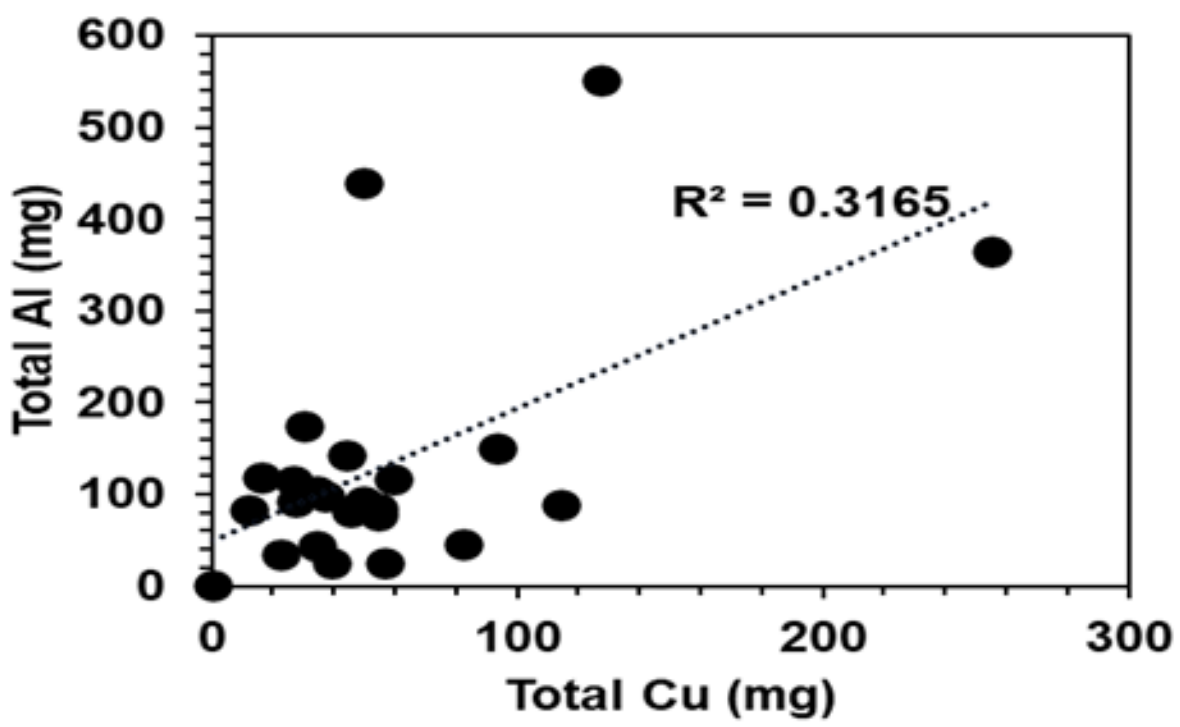

(o)

Figure S11. Correlation between masses of total extracted metals Zn & Fe (a), Al & Zn (b), Zn & Cu (c), Zn & Pb (d), Mn & Zn (e), Al & Pb (f), Mn & Al (g), Cu & Pb (h), Fe & Pb (i), Mn & Pb (j), Mn & Cu (k), Mn & Fe (l), Al & Fe (m), Fe & Cu (n), and Al & Cu (o).

Section S9. Total metals extracted versus cumulative water flow through filters.

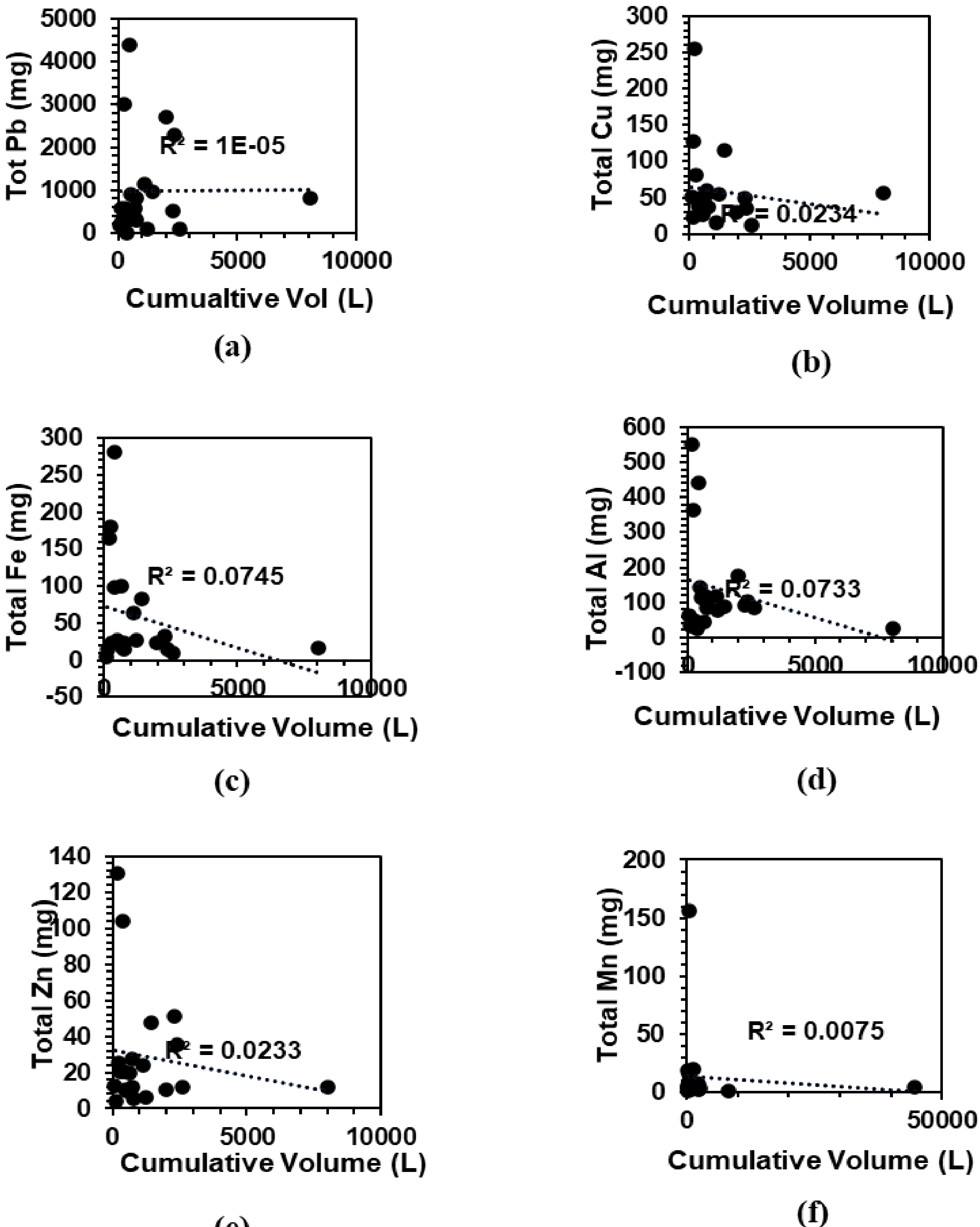

Figure S12. Total masses extracted of Pb (a), Cu (b), Fe (c), Al (d), Zn (e), and Mn (f) versus cumulative volume of water flow through filters
